# A crowd-sourcing approach for the construction of species-specific cell signaling networks

**DOI:** 10.1093/bioinformatics/btu659

**Published:** 2014-10-07

**Authors:** Erhan Bilal, Theodore Sakellaropoulos, Challenge Participants, Ioannis N. Melas, Dimitris E. Messinis, Vincenzo Belcastro, Kahn Rhrissorrakrai, Pablo Meyer, Raquel Norel, Anita Iskandar, Elise Blaese, John J. Rice, Manuel C. Peitsch, Julia Hoeng, Gustavo Stolovitzky, Leonidas G. Alexopoulos, Carine Poussin

**Affiliations:** ^1^IBM Research, Computational Biology Center, Yorktown Heights, NY 10598, USA, ^2^ProtATonce Ltd, Scientific Park Lefkippos, Patriarchou Grigoriou & Neapoleos 15343 Ag. Paraskevi, Attiki, Greece, ^3^National Technical University of Athens, Heroon Polytechniou 9, Zografou, 15780, Greece and ^4^Philip Morris International R&D, Philip Morris Products S.A., Quai Jeanrenaud 5, 2000 Neuchâtel, Switzerland

## Abstract

**Motivation:** Animal models are important tools in drug discovery and for understanding human biology in general. However, many drugs that initially show promising results in rodents fail in later stages of clinical trials. Understanding the commonalities and differences between human and rat cell signaling networks can lead to better experimental designs, improved allocation of resources and ultimately better drugs.

**Results:** The sbv IMPROVER Species-Specific Network Inference challenge was designed to use the power of the crowds to build two species-specific cell signaling networks given phosphoproteomics, transcriptomics and cytokine data generated from NHBE and NRBE cells exposed to various stimuli. A common literature-inspired reference network with 220 nodes and 501 edges was also provided as prior knowledge from which challenge participants could add or remove edges but not nodes. Such a large network inference challenge not based on synthetic simulations but on real data presented unique difficulties in scoring and interpreting the results. Because any prior knowledge about the networks was already provided to the participants for reference, novel ways for scoring and aggregating the results were developed. Two human and rat consensus networks were obtained by combining all the inferred networks. Further analysis showed that major signaling pathways were conserved between the two species with only isolated components diverging, as in the case of ribosomal S6 kinase *RPS6KA1.* Overall, the consensus between inferred edges was relatively high with the exception of the downstream targets of transcription factors, which seemed more difficult to predict.

**Contact:** ebilal@us.ibm.com or gustavo@us.ibm.com.

**Supplementary information:** Supplementary data are available at *Bioinformatics* online.

## 1 INTRODUCTION

Unveiling the inner workings of cell signaling networks is one of the long-standing challenges of systems biology. Small-scale versions of these networks have been built edge by edge using classic laboratory techniques such as immunoprecipitation, which has resulted in a large body of literature describing various gene and protein interactions. Although successful in their initial scope, these methods do not scale up to the genome level and are difficult to combine into a larger network, because of the different contexts in which they were originally reported. Organism, cell type, experiment timing and other conditions are crucial for determining whether an edge exists in a signaling network.

The advent of large-scale assays that can simultaneously measure the activity of thousands of genes has circumvented these aforementioned issues by enabling purely data-driven methods to infer large-scale networks. Various algorithms have been developed, including models based on Bayesian networks (Perrin *et al.*, 2003), mutual information (Margolin *et al.*, 2006), regression (Bonneau *et al.*, 2006), neural networks (Xu *et al.*, 2004), Boolean networks (Mitsos *et al.*, 2009) and differential equations (Chen *et al.*, 1999). Despite these advances, there is no clear best method. Each method has strengths and limitations influenced by how the methodology addresses the fact that network inference is inherently an underdetermined problem in the majority of cases (Marbach *et al.*, 2012; De Smet and Marchal, 2010). However, it has been observed that the aggregation of different network inference methods generates high-quality robust results (Marbach *et al.*, 2012).

Efforts to catalog and compare network inference algorithms have occurred in the form of data prediction competitions such as the ones organized by the Dialogue for Reverse Engineering Assessments and Methods (DREAM) consortium (Stolovitzky *et al.*, 2007). DREAM challenges participants to reconstruct cell signaling networks from gene expression datasets. Predicted networks are then evaluated based on a subset of known interactions, or the complete network in cases where the corresponding gene expression data were generated *in silico* (i.e. simulated).

DREAM is part of a larger group of successful crowd-sourcing initiatives in systems biology alongside CASP [critical assessment of protein structure prediction (Moult *et al.*, 1995)], CAFA [critical assessment of function annotation (Radivojac *et al.*, 2013)], CAPRI [critical assessment of prediction of interactions (Janin *et al.*, 2003)], FlowCAP [critical assessment of automated flow cytometry data analysis techniques (Aghaeepour *et al.*, 2013)] and Foldit [predicting protein structure with a multiplayer online game (Cooper *et al.*, 2010)]. In the same spirit as these academic initiatives, sbv IMPROVER is a crowd-sourcing-based methodology for the verification of research in an industrial setting (Meyer *et al.*, 2012). In its second installment, it challenges the research community to solve four problems related to the translation of molecular biology findings between rat and human model systems (Rhrissorrakrai *et al.*, 2015).

**Fig. 1.**
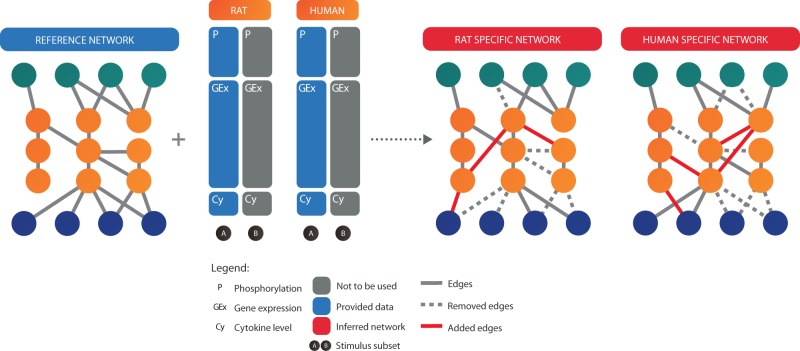
Overview of the Network Inference Challenge. Participants are provided with a reference network together with Affymetrix gene expression and Luminex phosphoproteomics and cytokine data derived from human and rat bronchial epithelial cells. The goal is to generate two separate networks for human and rat by adding and removing edges from the reference network using the data provided

Here we present the analysis of the Species-Specific Network Inference challenge, part of the sbv IMPROVER Species Translation set of challenges (https://www.sbvimprover.com). For this challenge, participants were asked to infer human- and rat-specific networks given phosphoprotein, gene expression and cytokine data (Fig. 1). The organizers also provided a common reference network from which participants had to generate the two networks by adding and removing edges. The purpose of this challenge was to augment and refine the reference map in a species-specific manner using data-driven approaches.

## 2 METHODS

### 2.1 Evaluation of inferred networks

Most of the prior knowledge regarding the interactions between elements in the cell signaling network was already incorporated in the reference map provided to the challenge participants. Hence, this information could not have been used as a gold standard against which to evaluate inferred networks. To circumvent this issue, we proposed that the true ranking of the submissions be viewed as a prediction problem in itself by combining different scoring strategies. Rank-based aggregation of individual predictions has been shown to provide robust results on par with the best performing methods in other data prediction challenges (Bilal *et al.*, 2013; Marbach *et al.*, 2012; Margolin *et al.*, 2013). Drawing from this result, the predicted networks were evaluated using softer methods that did not involve the use of a gold standard where the final ranks were derived by simply averaging the ranks obtained using the different scoring strategies.

The first scoring method involved the use of a published network inference algorithm (Mitsos *et al.*, 2009) to generate a ‘silver standard’ network against which all submissions were evaluated. This is mainly a pruning algorithm, hence only the subnetworks that intersected the reference network were scored. The following metrics were considered for this purpose: the z-score of the Jaccard similarity (JS), Matthews correlation coefficient (MCC) and the difference between the true-positive rate and false-positive rate (TPR-FPR) (Jaccard, 1912; Powers, 2007). In addition, two versions of the silver standard were generated: one that was trained on only the data available to the participants and one that also made use of part of the dataset that was kept hidden from participants and used as the gold standard in the other Species Translation challenges.

For another scoring method, the write-ups describing the methodology used for making the predictions were scored based on the following criteria: rigor, defined as the soundness of the proposed methodology based on valid statistics, arguments and premises without gaps in a logical, well-defined sequence of procedural steps; originality, defined as novelty in concept when compared with existing methods and typical approaches in the field; and practical implementation, defined as the ability to instantiate the proposed methods with existing or clearly described novel algorithms and commonly used computer architectures, the use of data sources commonly available to the field and a reasonable execution time. Three independent evaluators blindly assigned scores ranging from 1 (very poor) to 5 (very good) for each criterion, and then the final score was obtained by adding these points and then averaging among the evaluators.

### 2.2 The reference network

The reference network represents an ensemble of canonical pathways and was built following a top-down multi-layer hierarchical architecture starting with the stimulus layer through multiple signaling cascades to the transcription factor (TF) and secreted cytokine layers (Supplementary Fig. S2). Only stimuli with known mode of action present in subset A (training dataset) were included in the reference network.

The signaling cascade layer connected stimuli to latent (i.e. not measured) and measured (phosphoproteins) nodes representative of a membrane-to-nucleus protein signaling cascade (i.e., from stimuli to TF via kinase proteins). The identification and prioritization of latent nodes and edges (connectivity between stimuli, latent nodes and measured nodes) were conducted using various biological pathway databases (e.g. KEGG, Biocarta) and the ensemble network published by Kirouac *et al.* (2012), embodying the union of several online pathway databases. The network was traversed using a depth-first search algorithm, computing its transitive closure and identifying paths. Latent nodes that were not transitively connected to a stimulus or a measured node were removed. Additional latent nodes were identified based on topological features of the ensemble network. These highly connected nodes (counting the largest amount of incoming and outgoing connections) with a minimum overlap between them were identified using standard k-means clustering and integrated in the reference network.

For the TF layer, TFs corresponding to a subset of measured nodes and selected latent nodes were connected to a subset of target genes. These genes were identified using the Transcriptional Regulatory Element Database (Jiang *et al.*, 2007; Zhao *et al.*, 2005).

The cytokine layer was constructed by connecting target genes to corresponding measured cytokines. The final step included a manual verification and curation of the reference network to prune and refine it using literature reviews and various pathway databases (e.g. Biocarta, KEGG).

### 2.3 The silver standard network

The construction of the silver standard networks was based on a method developed by Mitsos *et al.* (Melas *et al.*, 2011; Mitsos *et al.*, 2009). The outline of this approach is to use Boolean logic to model signal transduction and integer linear programming (ILP) to fit the model to the data. In particular, Boolean logic was used to represent signal transduction in a prior knowledge network (i.e. reference network) to create a model capable of predicting the state of a node in a given experiment. Because Boolean models are limited to qualitative predictions, discretization of the experimental data was necessary. The discretization of the datasets was done by use of double threshold functions. In particular, the thresholds were set at ±2-fold changes for the gene expression data, ±3 standard residuals for the phosphoproteomics data and ±2 standard residuals for the cytokine data. The initial choice of thresholds was done in accordance with the processing methods used for the different data types as described in Poussin *et al.* (2014). In addition, a sensitivity analysis was performed to ensure that the final network would be robust on slight variations of the thresholds.

ILP was further used to combine the Boolean model with the experimental data by formulating an optimization problem that sought to minimize the mismatches between the predictions derived from the final network and the data at hand. The optimization procedure was performed by removing reactions from the reference network that were contradicted by the data and thus created a smaller data-specific network. More details about the silver standard model are available in the Supplementary Material.

### 2.4 The consensus network

The predictions from each of the *M* challenge participants can be organized as a binary vector $x_{j}=(x_{1j},x_{2j},...,x_{Nj})$ where j=1...M and *N* is the total number of possible edges, while the unknown gold standard network is represented as the vector $y=(y_{1},y_{2},...,y_{N})$. Each element $x_{ij}$ or $y_{i}$ can either be 1 (edge exists) or 0 (edge does not exist).

Let *P_T_* be the probability that a method predicts the existence of an edge given that the edge exists, and *P_F_* the probability of predicting the existence of an edge given that the edge does not exist. If $X_{i}$ and Y are random variables with realizations $x_{j}$ and y, respectively, then $X_{i}|Y=1$∼ *Bernoulli*(*P_T_*) and $X_{i}|Y=0$∼ *Bernoulli*(*P_F_*). Assuming that the predictions are independent given the true edge label, then the conditional distributions of the sum $X=X_{1}+X_{2}+...+X_{M}$ are modeled by the *Binomial* distributions:

(1) $$\mathrm{Pr}(X=k|Y=1)=\left(\begin{array}{c} M\\ k \end{array}\right)P_{T}^{k}{\left(1-P_{T}\right)}^{M-k}$$

(2) $$\mathrm{Pr}(X=k|Y=0)=\left(\begin{array}{c} M\\ k \end{array}\right)P_{F}^{k}{\left(1-P_{F}\right)}^{M-k}$$

where *k* is effectively the number of ‘votes’ received by an edge. Therefore, the probability density function that *k* teams picked the same edge is as follows:

(3) $$\mathrm{Pr}(X=k)=\frac{E}{N}\mathrm{Pr}(X=k|Y=1)+\frac{N-E}{N}\mathrm{Pr}(X=k|Y=0)$$

where *E* is the number of true edges.

The Equations (1) and (2) assume the performance of predictions is constant, modeled by parameters *P_T_* and *P_F_*; however, this is not true in practice. The variation in prediction performance between different algorithms can be modeled by imposing *P_T_* and *P_F_* to follow *Beta* distributions, normally used to model random variables limited to intervals of finite length. Consequently, the conditional probability functions in Equations (1) and (2) become the *Beta-Binomial* compound distributions:

(4) $$\mathrm{Pr}(X=k|Y=1)=\left(\begin{array}{c} M\\ k \end{array}\right)\frac{B(k+a_{1},M-k+b_{1})}{B(a_{1},b_{1})}$$

(5) $$\mathrm{Pr}(X=k|Y=0)=\left(\begin{array}{c} M\\ k \end{array}\right)\frac{B(k+a_{2},M-k+b_{2})}{B(a_{2},b_{2})}$$

where *P_T_* follows the beta distribution *B*(*a_1_,b_1_*) with shape parameters *a_1_* and *b_1_*_,_ and *P_F_* follows the beta distribution *B*(*a_2_,b_2_*) with shape parameters *a_2_* and *b_2_*.

The model described by the Equations (3), (4) and (5) can be fitted to the distribution of the data comprising the number of times each edge was present among the different proposed networks. An optimal consensus network can be reconstructed using all the predictions by finding the minimum number of votes per edge that satisfies the condition Pr(*Y = 1|X = k*) *>* Pr(*Y = 0|X = k*). This threshold can be easily found by numerically solving the following equation:

(6) $$\frac{\mathrm{Pr}(Y=1|X=k)}{\mathrm{Pr}(Y=0|X=k)}=\frac{\mathrm{Pr}(X=k|Y=1)\mathrm{Pr}(Y=1)}{\mathrm{Pr}(X=k|Y=0)\mathrm{Pr}(Y=0)}=1$$

where Pr(*Y = 1*) and Pr(*Y = 0*) are prior probabilities related to the true number of edges in the network:

(7) $$\mathrm{Pr}(Y=1)=\frac{E}{N}$$

(8) $$\mathrm{Pr}(Y=0)=1-\frac{E}{N}$$

## 3 RESULTS

The described methodology for building the reference network created a directed graph with 220 nodes and 501 edges organized into cascading layers where the edges are oriented from the top to the bottom layers. At the top is the stimulus layer that contains a subset of the compounds used to generate the training data, followed by receptor, adaptor, signaling, TF, target and cytokine layers (Supplementary Fig. S2). It is interesting to note that not all the TFs are reachable from all the stimuli nodes. The addition of a top stimuli layer to an otherwise generic network introduces the notion of context to pathways that are only active under certain conditions.

**Fig. 2.**
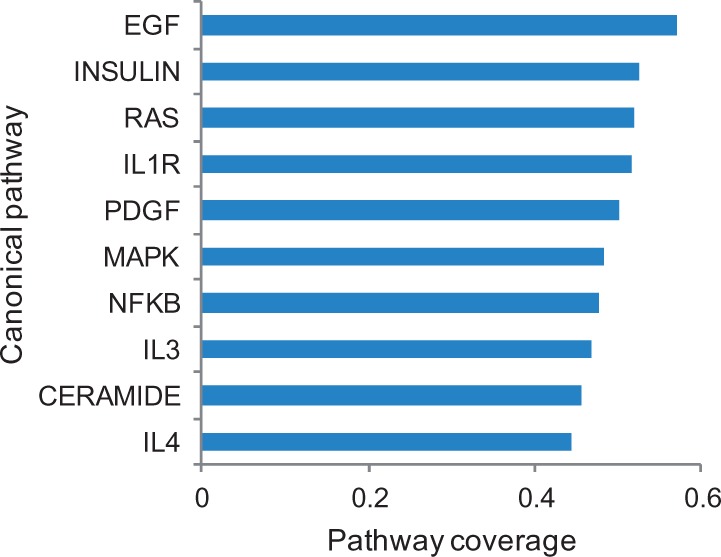
The top 10 canonical pathways represented in the reference network. The pathways are ordered by the proportion of genes present in the reference network

By mapping the nodes from the reference network to the genes from the canonical pathways listed in the Molecular Signature Database v3.1 (Subramanian *et al.*, 2005), we observe a diverse representation of cellular processes. Among the most common were cell growth and survival (*EGF*, *INSULIN*, *PDGF* and *RAS*), interleukin (*IL1R*, *IL3* and *IL4*), inflammatory response (*NFKB*) and cell signaling (*MAPK*) as shown in Figure 2.

### 3.1 Comparison of predicted networks

Challenge participants were allowed to add or remove edges from the reference network, although they were not allowed to add extra nodes. The purpose of this was to make submissions comparable and to put some boundaries that were relevant to the experiments performed. It is interesting to note that most proposed networks were built by removing edges from the reference network rather than adding additional interactions, which led to a bigger consensus on the existence of edges that were already part of the reference network. (Supplementary Figs S3 and S4). The median number of edges of the proposed networks was 406 for human and 429 for rat compared with 501 edges of the reference network.

In the case of the silver standard, two versions of the networks were considered: one that relied only on the training dataset and numbered 131 edges for human and 175 for rat, and one that used the full dataset (training and testing sets) and numbered 114 edges for human and 162 edges for rat. The JS between the two silver standards was 0.50 for human and 0.67 for rat. However, when using the two silver standard versions to evaluate the submissions, the scores obtained were very similar (Supplementary Table S1). This led to the decision to use only the first proposal, which used the same data as the challenge participants.

**Fig. 3.**
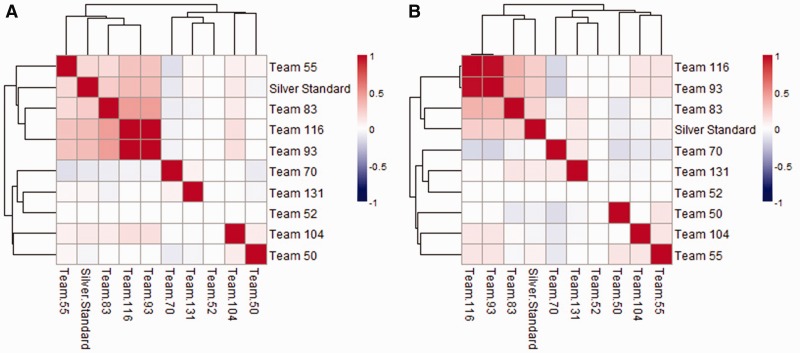
The predicted networks for human (**A**) and rat (**B**) were compared with the silver standard and against each other using MCC. Only edges present in the reference network were considered

The heatmaps in Figure 3 show the similarity between predicted networks together with the silver standard using MCC in the space of the reference network edges. Both panels suggest an emerging pattern where a few of the networks are more similar to each other and to the silver standard. The same can be observed when looking at the number of edges that overlaps between the different networks (Supplementary Tables S2 and S3). These are the networks that were ranked higher independent of the scoring metric used (i.e., JS, MCC or TPR-FPR) (Supplementary Table S4).

The second method for evaluating submissions used the scores obtained by the accompanying write-ups describing the algorithms used to build the species-specific networks. The scores listed in Supplementary Table S5 are separated by criterion (originality, rigor, practical implementation) and show remarkable consistency between reviewers. In the end, the final ranking was calculated by averaging the ranks obtained by each team for the two scoring methods and are listed in Supplementary Table S6.

All the predicted networks can be used to construct a consensus network by keeping the edges chosen by at least a predetermined number of teams. Supplementary Figure S5 shows how all the participating teams would have fared against a consensus network constructed using different thresholds from three to seven teams. Because two of the teams had similar entries (Fig. 3), one of them was discarded (Team 93) to avoid bias when the consensus network was built. It is worth noting that the top performing teams determined by consensus scoring using large thresholds (Teams 116 and 55) were the same as the ones that were the challenge best performers according to Supplementary Table S6. In contrast, the performance of lower ranked teams was less consistent between the different scoring strategies.

### 3.2 Optimal consensus network

**Fig. 4.**
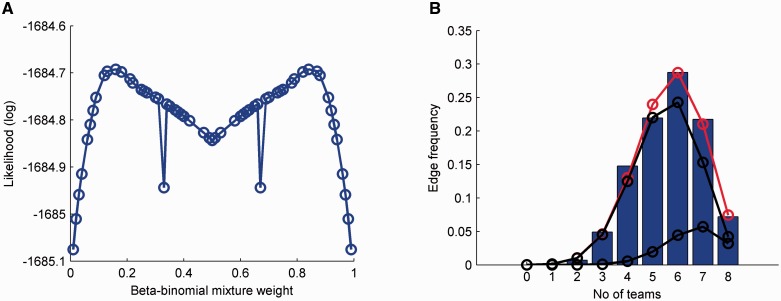
(**A**) The beta-binomial mixture weight can be calculated by maximizing the log-likelihood function. (**B**) Using this value, the fitted mixture is shown in red together with the individual-weighted components in black. Only edges present in the reference network were used in this case

The optimal threshold for building the consensus network was determined by fitting the model described in Section 2 (Equations 3, 4, and 5) followed by solving Equation 6. The data used for the fit were assembled by counting the number of ‘votes’ received by each edge in the reference network from the participating teams (excluding Team 93) and the silver standard network. This was performed separately for human and rat networks, and then the resulting datasets were mixed to improve the fit. Maximizing the log likelihood function of the mixture of two beta-binomial distributions (Equation 3) for different mixing constants led to Pr(*Y* = *1*)* = 0.16*, Pr(*Y* = *0*)* = 0.84* (Fig. 4A) and shape parameters *a_1_* = 8.77e+06, *b_1_* = 1.95e+06, *a_2_* = 3.46e+06 and *b_2_* = 1.57e+06. Using this result and after solving Equation 6, it was found that it takes approximately eight votes to verify the condition Pr(*Y* = *1|X* = *k*) *>* Pr(*Y* = *0|X* = *k*). This result can also be visualized in Figure 4B by tracing the intersection of the two mixture components depicted in black.

The model was tested on two additional datasets and showed a good overall fit (Supplementary Fig. S6). In the first case, the edge counts shown in Figure 4B were extended to all possible edges, including the ones not present in the reference network. In the second case, a completely new set of network predictions was obtained from DREAM 3 (Prill *et al.*, 2010). For this challenge, 27 participants had to predict *de novo* a synthetic network with 50 nodes and 82 edges from simulated gene expression data without knowing the identity of the nodes.

### 3.3 Conservation and divergence of human and rat cell signaling networks

**Fig. 5.**
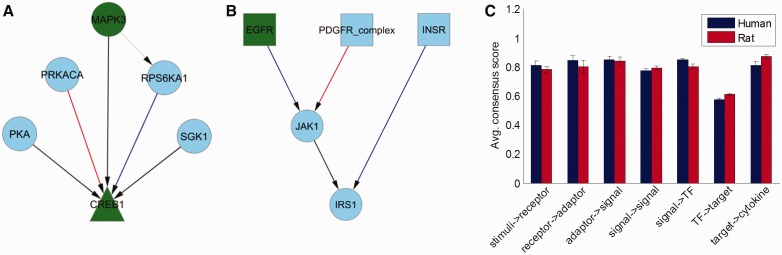
Panels A and B show two example subnetworks of the consensus network where in blue are human-specific edges, in red rat-specific edges and in black edges common to both species. Depicted in gray are edges from the original reference network that did not gather sufficient consensus between participants. Panel C shows the average consensus score of the edges between a layer and the next one downstream from it for human and rat networks

Using the threshold determined in the previous section, two consensus networks were built for human and rat using the networks predicted by participants together with the silver standard. The individual edges that resulted are depicted in Supplementary Figure S7 and color-coded based on their presence in the human, rat or both consensus networks. The number and the size of the resulting connected components are listed in Supplementary Table S7. Two of these subnetworks are shown in Figure 5 panels A and B as examples of predicted differences between human and rat cell signaling networks. Although there were plenty of edges that were active only in human or rat, these differences were rather isolated. The differences between human and rat did not scale up to the level of pathways or other higher levels of organization, as will be reinforced in the following analysis.

For any group of edges, a consensus score can be calculated by averaging the individual scores associated with each edge, which is simply the percentage of times the edge was predicted to exist. Here we assume that consensus between participants regarding an edge is associated with higher probability that the edge is real. The panel C in Figure 5 shows the average consensus scores of the edges between consecutive layers for human and rat together with the associated standard errors. Although there were no significant differences between human and rat, the overall consensus for the edges downstream of TFs seemed to be much lower than the rest, suggesting that these edges were more difficult to predict. The consensus scores of the edges in the canonical pathways listed in Figure 2 also showed no significant differences between human and rat (Supplementary Fig. S8A).

The conservation of phosphoprotein activity was measured by calculating the average consensus score of all edges adjacent to a phosphoprotein node (Supplementary Fig. S8B). From all the proteins measured, *RPS6KA1* had a significantly higher consensus score in human (*P*-value = 0.0161) and *WNK1* had a significantly higher consensus score in rat (*P*-value = 0.0498). Similarly, the conservation of TF activity was assessed by calculating the consensus score of the edges upstream of a TF (Supplementary Fig. S8C) and then downstream of it (Supplementary Fig. S8D). Edges upstream of *STAT1* had a higher consensus score in human than in rat (*P*-value = 0.0004), whereas edges downstream of *MYC* also showed a higher consensus score in human (*P*-value = 0.0287). Significantly higher consensus scores in rat were found for edges downstream of *TCF3*, *GLI2* and *SMAD3* (*P*-values = 8.8-e06, 0.0287 and 0.0156).

## 4 DISCUSSION

The scope of sbv IMPROVER Species Translation challenges was to assess the limits of using rat models to predict human biology in the specific context of bronchial epithelial cells exposed to various stimuli. Along these lines, the rationality behind the Network Inference challenge was to build two species-specific cell signaling networks starting from a generic literature-inspired network and using high-throughput proteomics and transcriptomics data to add or reject edges. This challenge differed from other challenges because it did not come with a gold standard (i.e. the true human and rat networks are unknown) and this posed difficulties in scoring and interpreting the results. The current work details how the aforementioned issues were addressed together with the lessons learned from organizing and curating such a challenge.

Despite the apparent top-down organization of the reference network, some feedback loops were present consistent with the structure of known pathways. However, the challenge experiments were designed to capture a broad area of the signaling network and not feedback mechanisms. The latter would have required a different experimental setup with more samples collected at later time points, as feedback loops tend to be more prominent at longer time scales.

Without a gold standard, individual scoring criteria can potentially be useful in separating poor performers from good performers, but can also have flaws. The silver standard is biased by the choice of algorithm used to generate it, and the quality of the write-ups does not always predict the best performing algorithms. It is thus advisable to combine the rankings resulting from individual scoring methods to reduce bias. The best performers obtained in this manner were the same as the ones obtained by comparing predictions with a consensus network built by aggregating the submissions from all participants. This result suggests that consensus scoring could be used as a legitimate scoring strategy for future challenges where a gold standard is absent.

The network aggregation procedure described in this article provides a statistically sound way of merging predicted networks or any other binary predictions given a sufficiently large sample space. This is especially useful when a clear way of assessing the best performing method is absent. However, even when one can accurately determine the best algorithm for performing a specific task, the result might be context dependent. It has been shown that disease classifiers vary greatly in performance when applied to different datasets (Tarca *et al.*, 2013). Aggregating multiple predictions has been proven to generate a more robust outcome on par with the best performing methods (Bilal *et al.*, 2013; Marbach *et al.*, 2012).

The generation of a consensus prediction can potentially have benefits beyond that of robustness and performance, particularly in the absence of a gold standard. The data shown in Supplementary Figure S5 suggest that predictions can be scored against a consensus network instead of using a silver standard, with similar top rankings when an appropriate threshold is used. Consensus scoring can thus avoid any bias caused by the choice of algorithm for the silver standard; however, it could be sensitive to outliers (e.g. predictions that are much better than the rest), or multiple correlated predictions caused by collaborating teams or the use of similar methods.

The predicted networks were aggregated using a mixture of two beta-binomial distributions as shown in Section 2. To find the optimal threshold for determining the existence of an edge, a two-step process was used. First, the distribution in Equation 3 was fitted to the consensus data; then the minimum number of teams *k* was determined for which Pr(*Y* = *1|X* = *k*) *>* Pr(*Y* = *0|X* = *k*). From the first step, the value of the mixture constant Pr(*Y = 1*) (Equation 7) can give an indication of the proportion of true edges in the reference network which in this case was 16%. Despite this, the solution to the second step resulted in consensus networks with 7.4% edges for human and 6.7% edges for rat out of all the reference network edges. This result suggests that less than half of the potential regulatory connections were discovered and more challenge participants were needed to increase statistical power and reconcile the two estimates of the number of true edges.

Despite these limitations, the consensus network shown in Supplementary Figure S7 displays some interesting patterns, some of which are shown in Figure 5A and B. Overall, the cAMP-responsive element-binding protein 1, also known as *CREB1*, showed the best consensus for the edges upstream of it (Supplementary Fig. S8C) but with a couple of differences between human and rat: the connection from *RPS6KA1* was present only in the human consensus network (Fang *et al.*, 2005), whereas the connection from *PRKACA* was present only in the rat consensus network (Wang *et al.*, 2006). The prevalence of *RPS6KA1* (a.k.a. *RSK1*) interactions in human (Supplementary Fig. S8B) might be explained by the fact that human isoforms of RSK1 have functional redundancy (i.e. *RPS6KA3* [*RSK2*]; *RPS6KA2* [*RSK3*]; and *RPS6KA6* [*RSK4*]). In contrast, this is most likely not the case in rodents; Zeniou *et al.* (2002) reported that the mouse *RSK1* and *RSK3* genes may not be able to fully compensate for the lack of *RSK2* function.

The consensus results also suggest a preference for *JAK1* activation through *EGFR* for human and the *PDGFR* complex for rat. Direct interaction between *JAK1* and *IRS1* has been reported in cultured human peripheral blood T cells (Johnston *et al.*, 1995). In rat, however, the interaction seems to be indirect through proteins *SOCS2*, *SOCS3* and *JAK2* (Calegari *et al.*, 2003; Park *et al.*, 2000). Other conserved interactions include *IFNGR1* to *JAK2* and *JAK2* to *STAT5A*, which are parts of the interferon gamma pathway known to be conserved across vertebrate species (Pestka *et al.*, 2004).

Additional references are provided for the majority of edges from the consensus networks and are available as Supplementary Material. These references are categorized by organism and tissue context as follows: airway cells, non-lung cells and lung cancer epithelial cells. Although numerous pathway databases are widely available, they are too generic and lack specific context when displaying an interaction. The purpose of this challenge was to fine tune one of these generic networks based on data collected from bronchial primary cells exposed to specific stimuli (compounds). When comparing the resulting consensus network to networks obtained from the Ingenuity Pathway Analysis tool (IPA: www.ingenuity.com), we observe a steady increase in precision as the number of votes required for an edge increases (Supplementary Fig. S9), culminating at eight votes as predicted by the model in Equation 6. The maximum precision obtained is 0.33 for the human network and 0.09 for the rat network. However, this could be explained by the relatively few edges identified in IPA for human (69) and especially rat (26) (the number of edges drastically decreased if a filter on cell/tissue type was applied), as well as the lack of proper context provided by tissue specificity and stimuli. The IPA networks as well as the consensus networks are available as Supplementary Material.

Overall, the fact that fewer suitable edge additions existed in most inferred networks (Supplementary Fig. S3) indicates that the reference network contains probably most of the true active pathways in both species. However, as observed by the large number of edge removals, it also contains many inactive pathways. In other words, the phosphoproteins represented by network nodes were less responsive to some stimuli than expected from the reference network. Furthermore, because most participants (and all top performers) used the reference network in their models it is likely that expert/prior knowledge was critical for optimal network construction.

The methods used by the participants to solve the challenge were varied and included Bayesian networks, Boolean networks, mutual information, lasso and elastic net, ANOVA and various heuristics (more details on the individual algorithms are available in the Supplementary Methods). It is interesting to note that different flavors of the same method, in this case Bayesian networks, do not perform similarly when applied to the same problem. When designing a prediction algorithm, a multitude of choices were made, ranging from various constants and priors to learning criteria and regularization options, which can lead to vastly different outcomes. This justifies efforts, such as the sbv IMPROVER challenges or any of the other crowd-sourcing initiatives such as DREAM or CASP, to try and establish best practices in the ever-changing field of computational biology.

## Supplementary Material

Supplementary Data
