# Supplementary material for: A crowd-sourcing approach for the construction of species-specific cell signaling networks
Source: Bioinformatics. 2014 Oct 7;31(4):484–91. doi: 10.1093/bioinformatics/btu659 (PMC4325542; doi:10.1093/bioinformatics/btu659)
Supplement: Supplementary Data [file supp_31_4_484__index.html]

A crowd-sourcing approach for the construction of species-specific cell signaling networks — A crowd-sourcing approach for the construction of species-specific cell signaling networks — A crowd-sourcing approach for the construction of species-specific cell signaling networks — Supplementary Data 

# A crowd-sourcing approach for the construction of species-specific cell signaling networks

## Supplementary Data

files

**Files in this Data Supplement:**

- Supplementary Data - doc file
- Supplementary Data - doc file
- Supplementary Data - xls file
- Supplementary Data - xls file
