# Supplementary material for: A crowd-sourcing approach for the construction of species-specific cell signaling networks
Source: Bioinformatics. 2014 Oct 7;31(4):484–91. doi: 10.1093/bioinformatics/btu659 (PMC4325542; doi:10.1093/bioinformatics/btu659)
Supplement: Supplementary Data [file supp_btu659_STC_SC4_paper_supp1.doc]

| A crowd sourcing approach for the construction of species specific cell signaling networks (Supplementary figures and tables)  Erhan Bilal^1, §,*^, Theodore Sakellaropoulos^2,4, §^, Challenge Participants^3^, Ioannis N. Melas^2,4^, Dimitris E. Messinis^2,4^, Vincenzo Belcastro^5^, Kahn Rhrissorrakrai^1^, Pablo Meyer^1^, Raquel Norel^1^, Anita Iskandar^5^, Elise Blaese^1^, John J. Rice^1^, Manuel C. Peitsch^5^, Julia Hoeng^5^, Gustavo Stolovitzky^1^, Leonidas G. Alexopoulos^2,4^ and Carine Poussin^5^  ^1^IBM Research, Yorktown Heights, NY 10598, USA  ^2^ProtATonce Ltd, Scientific Park Lefkippos, Patriarchou Grigoriou & Neapoleos 15343 Ag. Paraskevi,  Attiki, Greece  ^3^A complete list of the challenge participants is available in the Supplementary Information  ^4^National Technical University of Athens, Heroon Polytechniou 9, Zografou, 15780, Greece  ^5^Philip Morris International R&D, Philip Morris Products S.A., Quai Jeanrenaud 5, 2000 Neuchâtel,  Switzerland  ^§^Equal contribution |
| --- |


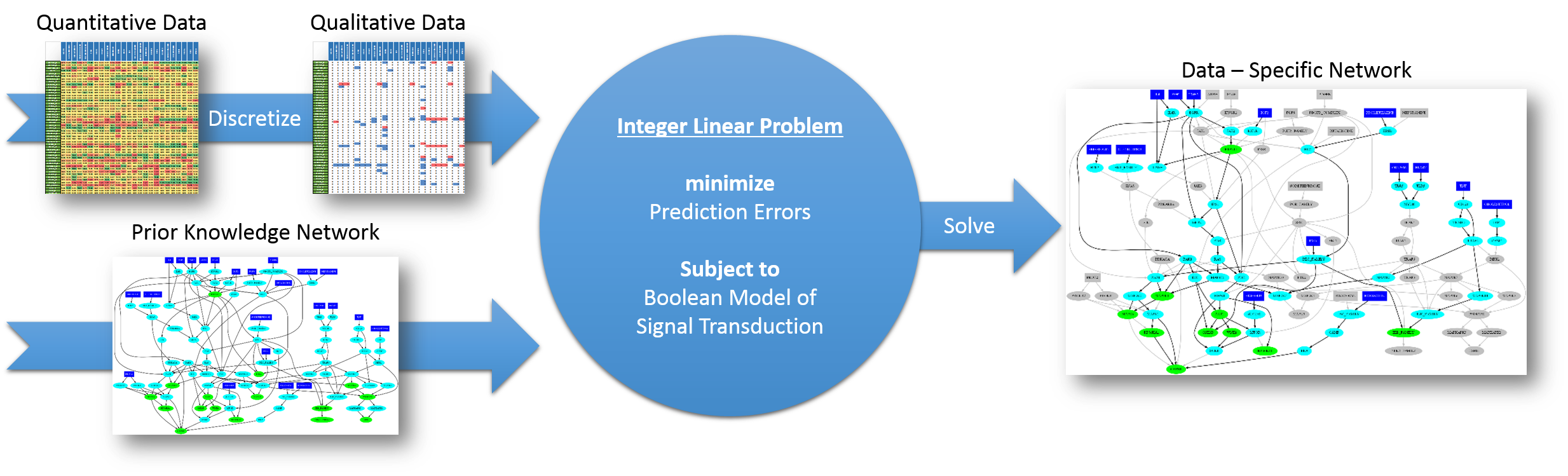


**Supplementary Fig. 1.** Schematic describing the process of generating the silver standard network.

**Supplementary Fig. 2.** The reference network is divided into cascading layers from stimuli to receptors, adaptors, signaling proteins, transcription factors, targets and cytokines.

**Supplementary Fig. 3.** The number of edges in the reference network (RN), predicted networks and silver standard for human (A) and rat (B).

**Supplementary Fig. 4.** Histograms showing the number of teams that picked an edge that was part of the reference network (A & B) or outside the reference network (C & D) for human (A & C) and rat (B & D).

**Supplementary Fig. 5.** Consensus networks are built by considering edges that are in common from 3 and up to 7 teams. The figure above shows how the teams would rank against the consensus network for different thresholds in human (A) and rat (B).

**Supplementary Fig. 6.** Beta-binomial mixture fits shown in log-scale for the Network Inference challenge submissions including edges not present in the reference network (A) and for a set of predictions taken from Dialogue for Reverse Engineering Assessments and Methods (DREAM) 3^rd^ edition challenge (B).

**Supplementary Fig. 7.** Consensus network obtained by combining participants' submissions together with the silver standard network. The edges that are common in both human and rat consensus networks are drawn in black, the ones that are exclusive to the human consensus network are drawn in blue and the ones that are exclusive to the rat consensus network are drawn in red. The rest of the edges depicted in light grey were not part of any consensus network but were part of the original reference network.

**Supplementary Fig. 8.** Average consensus scores (with error bars) of the edges in canonical pathways (A), adjacent to phospho-proteins (B), upstream of transcription factors (C) and downstream of transcription factors (D). Phospho-proteins and transcription factors with less than three associated edges were ignored.


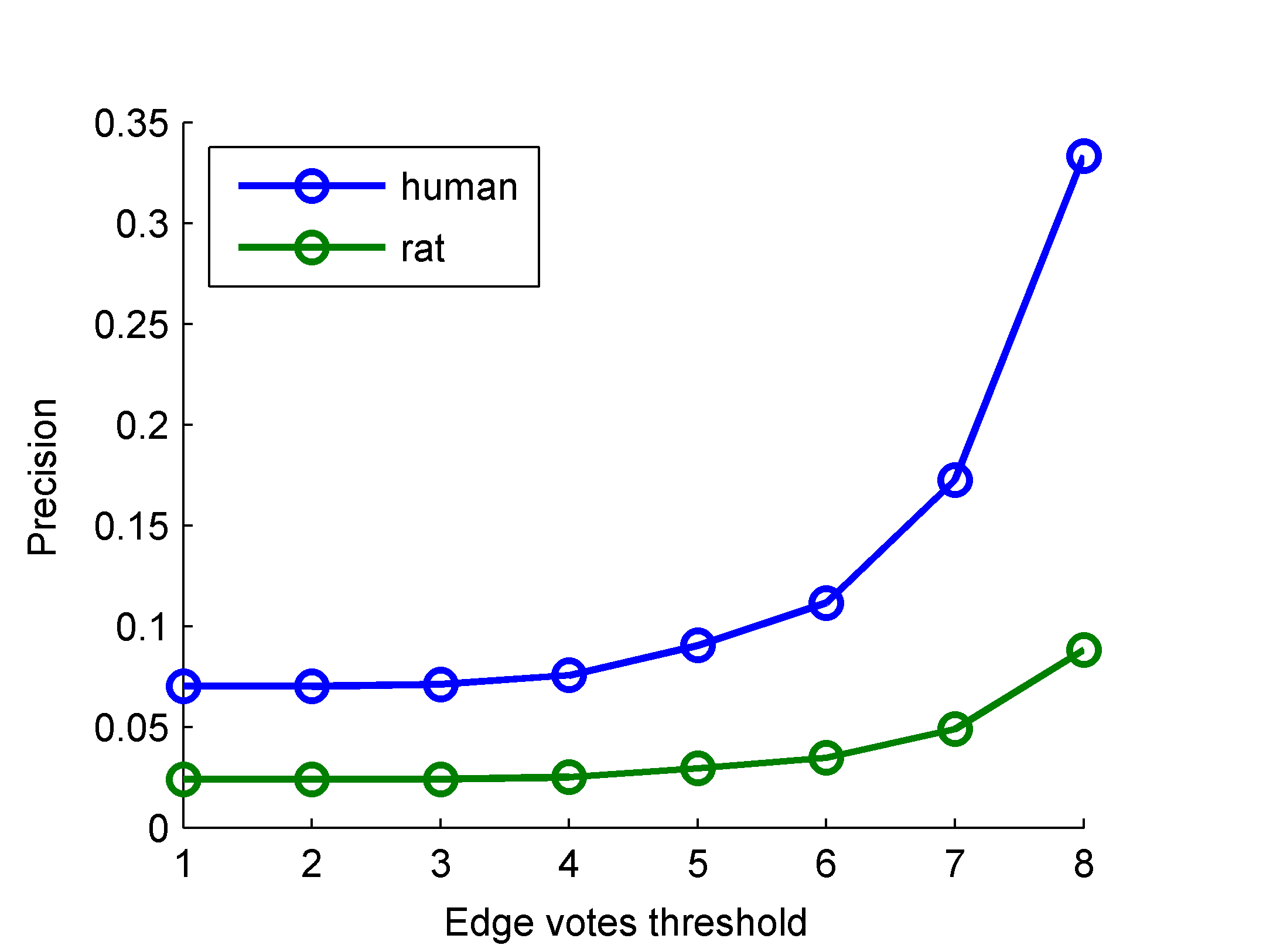


**Supplementary Fig. 9.** Precision of the human (blue) and rat (green) consensus networks calculated for different thresholds when compared to the IPA networks.


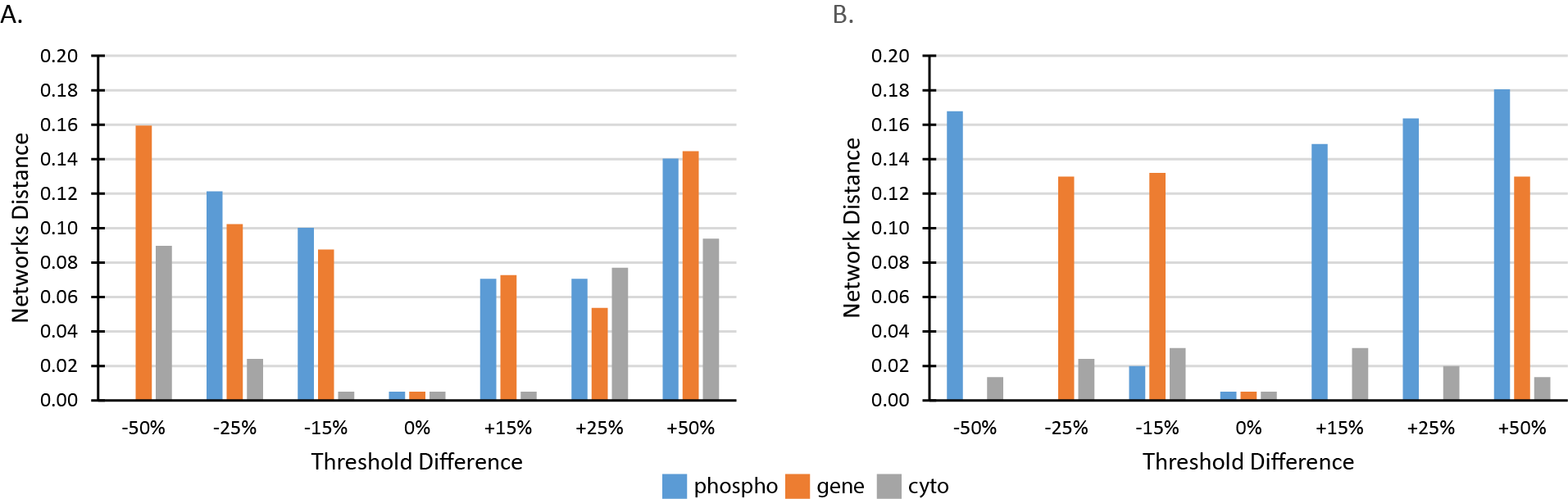
**Supplementary Fig. 10.** Results of sensitivity analysis for rat (A) and human (B). Changes in the threshold are depicted on the x axis as percentages of the original values while the distance of a network from the silver standard is depicted on the y axis. For every threshold change three different networks were computed, one for every dataset the change was imposed on and the results are depicted using different colors (blue for changes in the phosphoprotein dataset, orange for the gene expression dataset and grey for the cytokine dataset).

**Supplementary Fig. 11.**
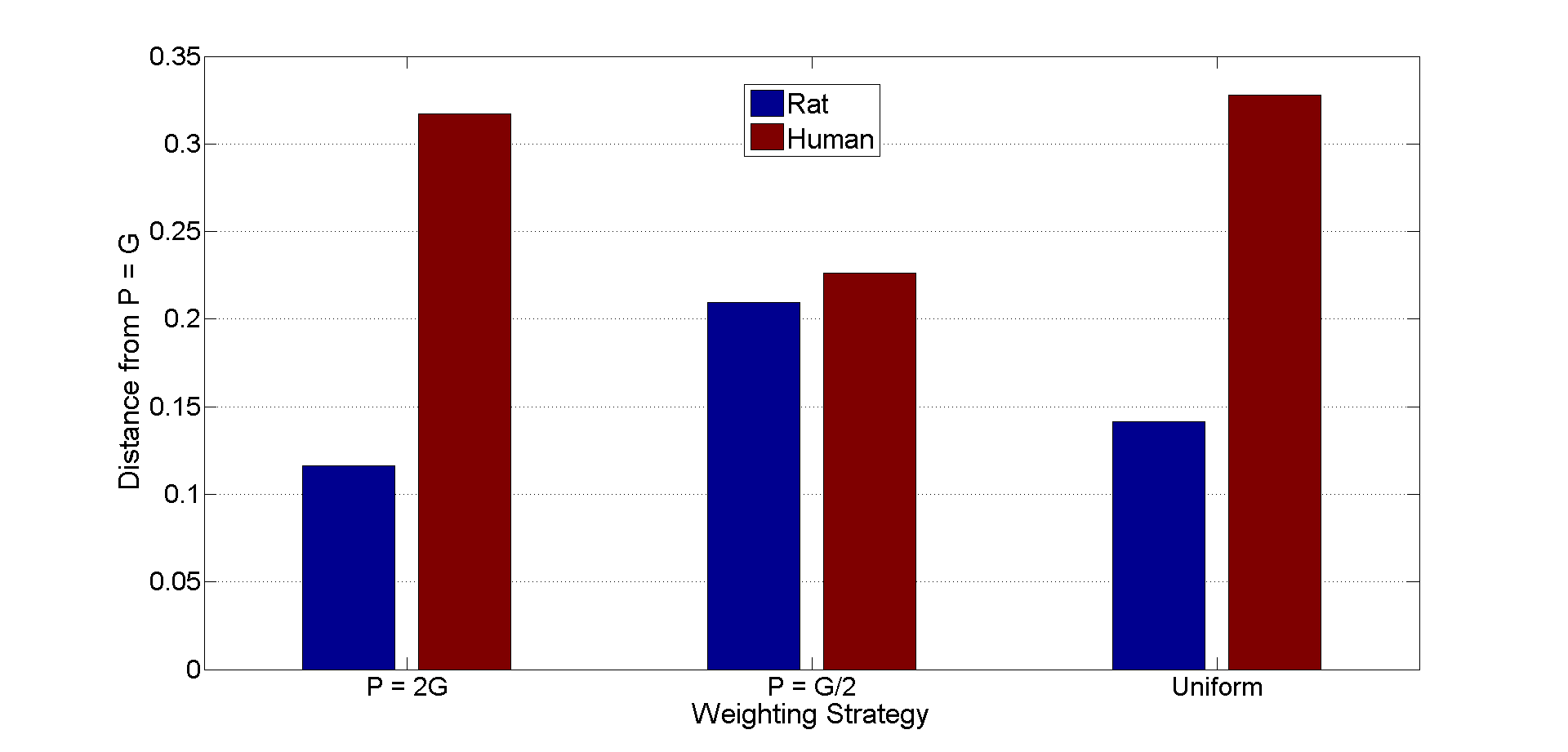
The weighting strategy for the different datasets (phosphoproteomic (P) and genomic (G)) was designed to attribute equal importance to the different experiments. Additional strategies considered are labeled on the *x* axis while the *y* axis shows the hamming distance of the resulting networks to the original network.


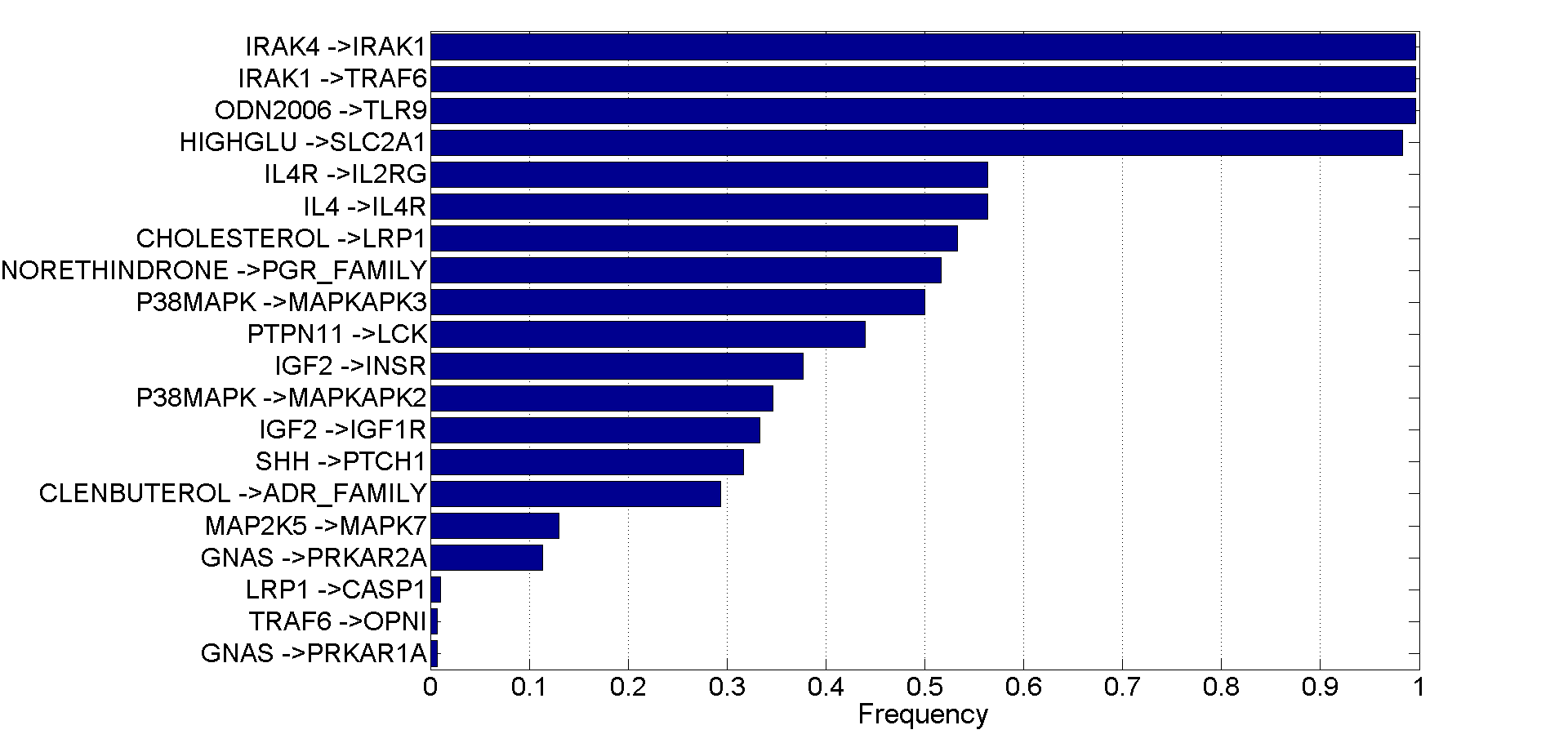


**Supplementary Fig. 12.** Out of 473 reactions present in the Rat silver standard network 20 vary between different optimal solutions. The *y* axis depicts these reactions together with the frequency with which they appear shown on the *x* axis.

**Supplementary Table 1.** Inferred networks scored against the silver standard generated using just the training data set or the full data set

| Team name | Training data set | | | Full data set | |
| --- | --- | --- | --- | --- | --- |
|  | Rat | Human | Rat | | Human |
| Team 104 | -0.021 | 0.043 | -0.038 | | 0.041 |
| Team 116 | 0.244 | 0.352 | 0.249 | | 0.341 |
| Team 131 | 0.029 | 0.008 | 0.03 | | 0.008 |
| Team 50 | -0.054 | -0.038 | -0.059 | | -0.006 |
| Team 52 | 0.000 | 0.000 | 0.000 | | 0.000 |
| Team 55 | 0.064 | 0.206 | 0.08 | | 0.247 |
| Team 70 | -0.014 | -0.102 | -0.016 | | -0.065 |
| Team 83 | 0.196 | 0.259 | 0.163 | | 0.201 |
| Team 93 | 0.238 | 0.352 | 0.252 | | 0.341 |

*Note:* The difference between true positive rate and false positive rate (TPR-FPR) was used as the scoring metric.

**Supplementary Table 2.** Number of edges that overlap between the human predicted networks, human silver standard and reference network. The last column shows the total number of edges in the respective network.

| Network | Team 104 | Team 116 | Team 131 | Team 50 | Team 52 | Team 55 | Team 70 | Team 83 | Team 93 | Silver Std. | Ref. Net. | Total edges |
| --- | --- | --- | --- | --- | --- | --- | --- | --- | --- | --- | --- | --- |
| Team 104 | 793 | 324 | 482 | 381 | 0 | 285 | 347 | 337 | 324 | 131 | 485 | 793 |
| Team 116 | 324 | 447 | 323 | 253 | 0 | 231 | 228 | 275 | 405 | 119 | 325 | 447 |
| Team 131 | 482 | 323 | 504 | 386 | 0 | 290 | 358 | 341 | 323 | 131 | 498 | 506 |
| Team 50 | 381 | 253 | 386 | 475 | 0 | 231 | 271 | 267 | 252 | 98 | 389 | 475 |
| Team 52 | 0 | 0 | 0 | 0 | 1 | 0 | 0 | 0 | 0 | 0 | 0 | 1 |
| Team 55 | 285 | 231 | 290 | 231 | 0 | 316 | 193 | 220 | 222 | 96 | 291 | 316 |
| Team 70 | 347 | 228 | 358 | 271 | 0 | 193 | 536 | 242 | 228 | 84 | 359 | 536 |
| Team 83 | 337 | 275 | 341 | 267 | 0 | 220 | 242 | 387 | 275 | 115 | 344 | 388 |
| Team 93 | 324 | 405 | 323 | 252 | 0 | 222 | 228 | 275 | 406 | 119 | 325 | 406 |
| Silver Std. | 131 | 119 | 131 | 98 | 0 | 96 | 84 | 115 | 119 | 131 | 131 | 131 |
| Ref. Net. | 485 | 325 | 498 | 389 | 0 | 291 | 359 | 344 | 325 | 131 | 501 | 501 |

**Supplementary Table 3.** Number of edges that overlap between the rat predicted networks, rat silver standard and reference network. The last column shows the total number of edges in the respective network.

| Network | Team 104 | Team 116 | Team 131 | Team 50 | Team 52 | Team 55 | Team 70 | Team 83 | Team 93 | Silver Std. | Ref. Net. | Total edges |
| --- | --- | --- | --- | --- | --- | --- | --- | --- | --- | --- | --- | --- |
| Team 104 | 706 | 326 | 470 | 373 | 0 | 303 | 304 | 369 | 328 | 151 | 480 | 706 |
| Team 116 | 326 | 465 | 321 | 257 | 0 | 226 | 192 | 288 | 415 | 131 | 327 | 465 |
| Team 131 | 470 | 321 | 511 | 379 | 0 | 304 | 318 | 380 | 323 | 160 | 491 | 513 |
| Team 50 | 373 | 257 | 379 | 477 | 0 | 256 | 234 | 290 | 256 | 118 | 388 | 477 |
| Team 52 | 0 | 0 | 0 | 0 | 12 | 0 | 0 | 0 | 0 | 0 | 0 | 12 |
| Team 55 | 303 | 226 | 304 | 256 | 0 | 337 | 187 | 234 | 218 | 106 | 310 | 337 |
| Team 70 | 304 | 192 | 318 | 234 | 0 | 187 | 454 | 241 | 191 | 101 | 321 | 454 |
| Team 83 | 369 | 288 | 380 | 290 | 0 | 234 | 241 | 429 | 288 | 144 | 384 | 429 |
| Team 93 | 328 | 415 | 323 | 256 | 0 | 218 | 191 | 288 | 420 | 131 | 329 | 420 |
| Silver Std. | 151 | 131 | 160 | 118 | 0 | 106 | 101 | 144 | 131 | 175 | 160 | 175 |
| Ref. Net. | 480 | 327 | 491 | 388 | 0 | 310 | 321 | 384 | 329 | 160 | 501 | 501 |

**Supplementary Table 4.** Silver standard ranking of the inferred networks using three different metrics

| Team name | JS^a^ | MCC^b^ | TPR-FPR^c^ |
| --- | --- | --- | --- |
| Team 104 | 6 | 6 | 6 |
| Team 116 | 1 | 1 | 1 |
| Team 131 | 5 | 5 | 5 |
| Team 50 | 8 | 9 | 9 |
| Team 52 | 9 | 6 | 6 |
| Team 55 | 4 | 4 | 4 |
| Team 70 | 7 | 8 | 8 |
| Team 83 | 3 | 3 | 3 |
| Team 93 | 2 | 2 | 2 |

*Note:* ^a^Jaccard similarity; ^b^Matthews correlation coefficient; ^c^Difference between true positive rate and false positive rate

**Supplementary Table 5.** Write-up scores

| Team name | Reviewer # | Originality | Rigor | PI^a^ | Total |
| --- | --- | --- | --- | --- | --- |
| Team 50 | 1 | 4.00 | 4.00 | 5.00 | 13.00 |
|  | 2 | 5.00 | 3.00 | 4.00 | 12.00 |
|  | 3 | 5.00 | 4.00 | 4.00 | 13.00 |
|  | **Average** | **4.67** | **3.67** | **4.33** | **12.67** |
| Team 52 | 1 | 2.00 | 3.00 | 3.00 | 8.00 |
|  | 2 | 1.00 | 1.00 | 3.00 | 5.00 |
|  | 3 | 2.00 | 2.00 | 4.00 | 8.00 |
|  | **Average** | **1.67** | **2.00** | **3.33** | **7.00** |
| Team 55 | 1 | 3.00 | 4.00 | 5.00 | 12.00 |
|  | 2 | 4.00 | 5.00 | 4.00 | 13.00 |
|  | 3 | 5.00 | 4.00 | 4.00 | 13.00 |
|  | **Average** | **4.00** | **4.33** | **4.33** | **12.67** |
| Team 70 | 1 | 1.00 | 1.00 | 4.00 | 6.00 |
|  | 2 | 3.00 | 2.00 | 3.00 | 8.00 |
|  | 3 | 2.00 | 2.00 | 4.00 | 8.00 |
|  | **Average** | **2.00** | **1.67** | **3.67** | **7.33** |
| Team 83 | 1 | 4.00 | 3.00 | 3.00 | 10.00 |
|  | 2 | 4.00 | 3.00 | 4.00 | 11.00 |
|  | 3 | 3.00 | 3.00 | 3.00 | 9.00 |
|  | **Average** | **3.67** | **3.00** | **3.33** | **10.00** |
| Team 93 | 1 | 3.00 | 4.00 | 4.00 | 11.00 |
|  | 2 | 3.00 | 3.00 | 4.00 | 10.00 |
|  | 3 | 3.00 | 5.00 | 4.00 | 12.00 |
|  | **Average** | **3.00** | **4.00** | **4.00** | **11.00** |
| Team 104 | 1 | 3.00 | 4.00 | 4.00 | 11.00 |
|  | 2 | 4.00 | 4.00 | 5.00 | 13.00 |
|  | 3 | 4.00 | 4.00 | 5.00 | 13.00 |
|  | **Average** | **3.67** | **4.00** | **4.67** | **12.33** |
| Team 116 | 1 | 2.00 | 3.00 | 3.00 | 8.00 |
|  | 2 | 3.00 | 4.00 | 4.00 | 11.00 |
|  | 3 | 4.00 | 3.00 | 3.00 | 10.00 |
|  | **Average** | **3.00** | **3.33** | **3.33** | **9.67** |
| Team 131 | 1 | 2.00 | 2.00 | 3.00 | 7.00 |
|  | 2 | 3.00 | 3.00 | 2.00 | 8.00 |
|  | 3 | 2.00 | 2.00 | 4.00 | 8.00 |
|  | **Average** | **2.33** | **2.33** | **3.00** | **7.67** |

*Note:* ^a^Practical implementation

**Supplementary Table 6.** Final ranking

| Team name | TPR-FPR rat score | TPR-FPR human score | TPR-FPR combined rank | Write-up score | Write-up rank | Final rank |
| --- | --- | --- | --- | --- | --- | --- |
| Team 55 | 0.064 | 0.206 | 4 | 12.67 | 1 | 1 |
| Team 116 | 0.244 | 0.352 | 1 | 10.33 | 4 | 1 |
| Team 93 | 0.238 | 0.352 | 2 | 10.33 | 4 | 3 |
| Team 83 | 0.196 | 0.259 | 3 | 10.00 | 6 | 4 |
| Team 104 | -0.021 | 0.043 | 6 | 12.33 | 3 | 4 |
| Team 50 | -0.054 | -0.038 | 9 | 12.67 | 1 | 6 |
| Team 131 | 0.029 | 0.008 | 5 | 7.67 | 7 | 7 |
| Team 52 | 0.000 | 0.000 | 6 | 7.00 | 9 | 8 |
| Team 70 | -0.014 | -0.102 | 8 | 7.33 | 8 | 9 |

*Note:* The second and third columns show the silver standard TPR-FPR scores of the corresponding teams for the rat and human networks. The combined rank in the fourth column is obtained by averaging the ranks of the rat and human scores from the second and third columns. The fifth and sixth columns show the write-up scores and the corresponding rank, while the last column lists the final rank obtained by averaging the combined silver standard ranks from column four with the write-up ranks from column six.

**Supplementary Table 7.** Connected components of the consensus networks

| Connected component size | Human consensus network | Rat consensus network |
| --- | --- | --- |
| 2 | 13 | 8 |
| 3 | 1 | 6 |
| 5 | 2 | 0 |
| 8 | 2 | 2 |
| Total | 18 | 16 |

*Note:* Each cell shows the number of the connected components of a given size.
