# Supplementary material for: A crowd-sourcing approach for the construction of species-specific cell signaling networks
Source: Bioinformatics. 2014 Oct 7;31(4):484–91. doi: 10.1093/bioinformatics/btu659 (PMC4325542; doi:10.1093/bioinformatics/btu659)
Supplement: Supplementary Data [file supp_btu659_STC_SC4_paper_supp2.doc]

| A crowd sourcing approach for the construction of species specific cell signaling networks (Supplementary methods)  Erhan Bilal^1, §,*^, Theodore Sakellaropoulos^2,4, §^, Challenge Participants^3^, Ioannis N. Melas^2,4^, Dimitris Messinis^2,4^, Vincenzo Belcastro^5^, Kahn Rhrissorrakrai^1^, Pablo Meyer^1^, Raquel Norel^1^, Anita Iskandar^5^, Elise Blaese^1^, John J. Rice^1^, Manuel C. Peitsch^5^, Julia Hoeng^5^, Gustavo Stolovitzky^1^, Leonidas G. Alexopoulos^2,4^ and Carine Poussin^5^  ^1^IBM Research, Yorktown Heights, NY 10598, USA  ^2^ProtATonce Ltd, Scientific Park Lefkippos, Patriarchou Grigoriou & Neapoleos 15343 Ag. Paraskevi,  Attiki, Greece  ^3^A complete list of the challenge participants is available in the Supplementary Information  ^4^National Technical University of Athens, Heroon Polytechniou 9, Zografou, 15780, Greece  ^5^Philip Morris International R&D, Philip Morris Products S.A., Quai Jeanrenaud 5, 2000 Neuchâtel,  Switzerland  *Challenge participants:* Gyan Bhanot^1^, Michael Biehl^2^, Dragan Bošnački^3^, Boris Breuer^3^, Chunhui Cai^4^, Anastasia Chasapi^5^, Lujia Chen^4^, Jie Cheng^6^, Adel Dayarian^7^, Julien Dorier^5^, Huub ten Eikelder^3^, Robert J. Flassig^8^, Nicolas Guex^5^, Sandra Heise^8^, Peter Hilbers^3^, Sahand Hormoz^7^, Mark Ibberson^5^, Steffen Klamt^8^, Xinghua Lu^4^, Jason McDermott^9^, Hugh Mitchell^9^, Joel G. Pounds^9^, Regina Samaga^8^, Susan Tilton^9^, Leonore Wigger^5^, Ioannis Xenarios^5^.  ^1^Department of Physics and Astronomy, Rutgers University, Piscataway, New Jersey, USA  ^2^Johann Bernoulli Institute for Mathematics and Computer Science, University of Groningen, Groningen, Netherlands  ^3^Eindhoven University of Technology, Eindhoven, Netherlands  ^4^Department of Biomedical Informatics, University of Pittsburgh, Pittsburgh, Pennsylvania, USA  ^5^SIB Swiss Institute of Bioinformatics, Center of Integrative Genomics, UNIL, Switzerland  ^6^Quantitative Sciences, GlaxoSmithKline, Collegeville, Pennsylvania, USA  ^7^Kavli Institute for Theoretical Physics, University of California Santa Barbara, Santa Barbara, California, USA  ^8^Max Planck Institute for Dynamics of Complex Technical Systems, Magdeburg, Germany  ^9^Pacific Northwest National Laboratory, Richland, Washington, USA |
| --- |

# Supplementary methods

# Data generation

The data sets used for this challenge consisted of phosphoproteomics, cytokine and gene expression measured in normal human and rat bronchial epithelial cells (NHBE and NRBE). The cells were exposed in parallel to 52 different compounds or to normal growth medium used as negative control (DMEM: Dulbecco's Modified Eagle's Medium) and then lysed at different time points depending on the type of measurement performed. Phosphoproteins were measured in cell lysates collected at 5 and 25 minutes after adding the stimulus (compound); gene expression was measured after 6 hours and cytokines were measured in supernatants collected after 24 hours. The exposure of cells to each stimulus was performed in triplicate and in four-to-six replicates for the DMEM control.

The time points were chosen based on established time scales appropriate for each reaction type. In the case of the phosphoproteomics data, preliminary experiments were performed that included five time points (0, 5, 15, 20 and 25 min). The time points at 5 and 25 minutes were selected based on the number and the magnitude of the phosphorylation events observed in one or both cell types. In the case of the transcriptomics and cytokine experiments, the choice of the time points was based on existing literature (Lam*b et a*l., 2006; Merten*s et a*l., 2010; Lalake*r et a*l., 2009).

The phosphoproteomics and cytokine experiments were performed using Luminex xMAP technology and involved measuring 19 phosphoproteins and 22 cytokines from cells exposed to the different compounds. The corresponding gene expression levels were measured using the Affymetrix HG-U133 Plus2 platform for human cells and Rat 230 2.0 for rat cells and processed using GCRMA (Zhijin Wu, 2004). More details on the experimental setting and data preprocessing can be found in Poussin et al. (Poussi*n et a*l., 2014).

## Approximately half of the data generated for the Species Translation (ST) challenge was made available to the participants as training data whereas the other half was kept hidden from participants because it was used as the gold standard in the other ST sub-challenges 1, 2 and 3 : https://sbvimprover.com/challenge-2/challenge-2-challenge.

# The Silver Standard network

## Model formulation

Assume a signaling network $G$ defined as a set of reactions indexed by $i=1,...,n_{r}$ and species indexed by $j=1,...,n_{s}$. Each reaction $i$ is defined by three index sets; the set of reactants $R_{i}$, the set of inhibitors $H_{i}$ and set of products $P_{i}$; such that $R_{i},H_{i},P_{i}\subseteq\{1,...,n_{s}\}$. We also define a set of experiments indexed by $k=1,...,n_{e}$. In each experiment a set of species $I_{j}^{k}\in\{0,1\}$ is perturbed. $I_{j}^{k}=1$ if species $j$ in experiment $k$ is used as a stimulus; and $I_{j}^{k}=0$ if it is not. Moreover, in each experiment a set of species are measured $M_{j}^{k}\in\{0,1\}$. $M_{j}^{k}=1$ if species $j$ in experiment $k$ is measured; $M_{j}^{k}=0$ otherwise. We also define variables $x_{j}^{k}\in\{0,1\}$ to denote the activation state of species $j$ in experiment $k$. $x_{j}^{k}=1$ if species $j$ is active in experiment $k$; $x_{j}^{k}=0$ otherwise. Finally we introduce variables $z_{i}^{k}\in\{0,1\}$ to denote whether reaction $i$ is active ($z_{i}^{k}=1$) or not ($z_{i}^{k}=0$) in experiment $k$ and variables $y_{i}\in\{0,1\}$ to denote whether a reaction $i$ is present ($y_{i}=1$) or not ($y_{i}=0$) in the final network.

Starting from the stimuli, the signal is propagated downstream following the rules of Boolean logic. Thus,

1. A reaction $i$ will only take place ($z_{i}^{k}=1$) if it is present in the network ($y_{i}=1$)
2. A reaction $i$ will take place in experiment $k$ ($z_{i}^{k}=1$) if all reactants are present (i.e. $x_{j}^{k}=1; \forall j\in R_{i}$) and no inhibitors are present (i.e. $x_{j}^{k}=0; \forall j\in H_{i}$).
3. If a reaction $i$ takes place all downstream species will be activated (i.e. $x_{j}^{k}=1; \forall j\in P_{i};$)
4. A species $j$ may only be active (i.e. $x_{j}^{k}=1$) if reaction $i$ exists and takes place (i.e. $z_{i}^{k}=1$) where this species is a product (i.e. $j\in P_{i}$); otherwise $j$ will be inactive (i.e. $x_{j}^{k}=0$).

The rules above may be formulated as linear constraints in the following manner:

$$x_{j}^{k}\geq I_{j}^{k};\forall j,k$$

$$z_{i}^{k}\leq y_{i};\forall j,k$$

$$z_{i}^{k}\leq x_{j}^{k};\forall i,j,k\in R_{i}$$

$$z_{i}^{k}\leq1-x_{j}^{k};i,j,k\in H_{i}$$

$$z_{i}^{k}\geq y_{i}+\sum_{j\in R_{i}} (x_{j}^{k}-1)-\sum_{j\in H_{i}} x_{j}^{k};\forall i,k$$

$$x_{j}^{k}\geq z_{i}^{k};i,j,k\in P_{i}$$

$$x_{j}^{k}\leq\sum_{i,j\in P_{i}} z_{i}^{k}+I_{j}^{k};\forall j,k$$

The Objective function of the problem is to minimize the prediction errors over all the measurements by removing from the Prior Knowledge Network as few reactions as possible.

$$\min_{x,y,z} \sum_{k} \sum_{j|M_{j}^{k}=1} w_{j}^{k}|x_{j}^{k}-x_{j}^{k,m}|-a_{i}y_{i}$$

where $x_{j}^{k,m}$ is the measured value of the species $j$ in the experiment $k$ and $w_{j}^{k}$, $a_{i}$ are weights.

## Data discretization

The thresholds chosen for the different data sets and normalizing techniques used represent typical choices: ±2 fold-changes for the gene expression data, ±3 standard residuals for the phosphoproteomics data and ±2 standard residuals for the cytokine data. In the case of the phosphoproteomics data a more relaxed threshold lead to more inconsistencies between the activation status of phosphoproteins amongst technical replicates, while a higher threshold for the cytokine data lead to fewer than 2% active cytokines.

A sensitivity analysis was performed to evaluate the robustness of the resulting networks with respect to the chosen thresholds. In this analysis the different thresholds were perturbed one at a time, followed by computing the distance of the resulted networks to the initial choice. Every network was represented as a binary vector of length equal to the number of reactions present in the reference network, where every element of the vector represented a decision variable indicating whether the corresponding reactions were included in the final network or not. The Hamming distance was used as a similarity metric between the resulting network vectors (i.e., the proportion of edits required to make the two vectors identical).

Due to the complexity of Integer programming (NP-hard) some of the simulations were computationally intractable. In consequence, the resulting networks for some cases could not be computed within the required precision (these cases appear as missing bars in Supplementary Fig. 10). From the cases that could be computed, Supplementary Fig. 9 shows that the method is slightly sensitive to changes in the data (hamming distance < 0.2). However, changes in the threshold appear to be proportional to the changes in the network distance indicating that the method is relatively robust to small perturbation; although the structure of the data does play a role, as the rat networks appear to be less sensitive to perturbation than the human ones.

## Parameter tuning

There are 2 types of user defined parameters involved in the ILP formulation:

- One ($w_{j}^{k}$) corresponding to the cost of a mismatch between the predicted and the measured value of a node at a given experiment and
- Another ($a_{i}$) corresponding to the gain of including a reaction in the final network.

In defining these parameter, the following requirements were considered:

1. The primary goal of the algorithm should be to match the experimental data, i.e. the cost of mismatching a measured value should not be balanced by the gain of including any number of reactions.

$$\sum_{i} a_{i}=\min(w_{j}^{k})$$

1. Because the different datasets used had significantly different sizes, selecting a uniform distribution of weights would have resulted in a final networked heavily biased primarily in favor of the Gene Expression dataset (and also for of the Cytokine dataset, since cytokines were mostly connected directly to single genes in the reference network). However, genes and cytokines were adjacent to only 72 out of the 501 reaction in the network. To account for these imbalances, we required the total cost of the phosphoprotein dataset (P) to be equal to the total cost of the gene expression (G) and cytokine (C) dataset, in order to force the algorithm to attribute equal importance to the different dataset.

$$\frac{\sum_{j\in P} w_{j}^{k}}{\sum_{j\in G\vee C} w_{j}^{k}}=1$$

1. Finally, in deciding the individual weights of each node at every experiment, the maximum entropy principle was enforced and every group ($a_{i}$,$w_{j\in P}^{k}$,$w_{j\in G\vee C}^{k}$) had a uniform distribution of weights.

The same weighting strategy was used for both the human and rat networks and different experiments were attributed equal importance – genomic (G) and phosphoproteomic (P). This was achieve by balancing the cost of all the phosphoproteomic measurements with the cost of all the genomic ones (P = G) during the optimization process. In order to evaluate the effect of the different weighting strategies other models were considered: equal weight for every measurement (uniform weighting), doubling the importance of the phosphoproteomic dataset (P=2G), and doubling the importance of the genomic dataset (P = G/2). The effects of the different weighting strategies are quantified in Supplementary Fig. 11 as the hamming distance of the resulting network from the silver standard network. The average hamming distance is around 0.2 which points to the conclusion that different weighting strategies can induce a moderate effect on the resulting network.

The terminating criterion for both the human and the rat silver standard was for the ILP optimality gap (i.e. the gap between the integer and the corresponding relaxed-continuous problem) to be lower than 5%. Both networks reached the required criterion however solving the problem was computationally very taxing especially for the human dataset which required more than 48 hours. Because of this limitation, we couldn’t generate multiple solutions. However, since algorithm was incentivized to remove as few reactions as possible, the only reactions removed were those that had been contradicted by the data. Therefore, the resulting network included all the reactions that could have possibly been transduced by signals, and thus would have been present in any solution, plus some extra reactions that could not be traced back to any stimulus and were removed afterwards through a simple graph traversal. In this way, the number of reactions that were falsely removed was minimized at the cost of including some possibly redundant reactions, which however could not be ruled out in the given framework.

The optimization process described does not always lead to a unique solution hence multiple simulations were run to assess the effect on the resulting network. After 300 runs using the rat datasets and previously described parameters, 20 reactions (<5%) were found to be unstable out of 473 total. These reactions are listed in Supplementary Fig. 12 together with the frequency with which they appear during the simulations.

## Team 55 (Steffen Klamt, Robert J. Flassig, Sandra Heise, Regina Samaga)

In order to derive species-specific network topologies from experimental data, we applied a recently published method (Mela*s et a*l., 2013) that is based on analyzing ***sign consistencies*** between a given reference network topology and experimental data (the latter obtained from perturbation experiments). The idea behind this approach is to change the network topology (by adding and removing edges to/from the reference network) in order to maximize consistency between network-based predictions and observed changes in measurement signals.

Our method requires two steps:

(1) Data processing (discretization) to classify observed effects of stimuli on the readout nodes as upregulation (+1), downregulation (-1), or no regulation (= unchanged activation level; 0).

(2) Use these discretized data to train a given reference network such that mismatches between observed and predicted node changes are minimized.

1. Data processing / Discretization

Measurements of node activities are binned or classified as upregulation, downregulation or no regulation (unchanged activation level) encoded as 1, -1 or 0, respectively. Non-measured nodes of the network are marked as such, for example with NaN. Classification for each data set is based on significant deviations from the unperturbed case. Nodes that had inconsistencies in the signed changes in the replicates or at the different time points are marked as NaN. In the following, details on the classification for each data set are given.

- 1. Protein phosphorylation level

The phosphorylation level $x_{Pi,j,k,t}$of protein *Pi* (*Pi* ∈ {1…17}) was measured in 3 replicates *k* (*k* ∈ {1,2,3}) per stimuli *j* (*j* ∈ {1..26}) at *t*=5 and *t*=25 minutes. The stimuli were distributed on two batches *b* (*b* ∈ {1,2}; the corresponding batch for stimuli *j* is indicated by *b(j)*) with controls for each protein for each batch. For each batch, we used the sample mean over all controls of protein *Pi* ($\bar{C}_{Pi,b(j),t}$) and the sample standard deviation of measurements of *Pi* over all stimuli from the same batch ($\sigma_{Pi,b(j),t}$) to generate the z-score for t=5 and t=25:

$Zscore_{Pi,j,k,t}=(x_{Pi,j,k,t}-\bar{C}_{Pi,b(j),t})/\sigma_{Pi,b(j),t}$.

The z-score is used for classifying potential regulations. When a z-score was bigger/smaller than a given threshold (here: thr=1/-1) we assumed a significant positive (+1) / negative (-1) effect, otherwise a 0. However a *consistent* pos./neg. effect by the perturbation was only assumed, if at least 2 of the 3 replicates showed this pos./neg. behavior for the same time point *t*. Measurements without significant changes, or with just one change out of 3 replicates have been classified as unchanged, i.e. binned to 0. If there was at least one positive and at least one negative effect in replicates for the same stimuli, the effect was inconsistent and is thus classified as NaN, i.e. the measurement is not accounted for.

The classification was done for the two time points at 5 and 25 minutes, which were then compared: Here again, if at least one of the two time points showed a positive/negative effect the result was finally classified as 1/-1. If none of the 5/25 minutes data sets showed an effect the effect of the perturbation on a specific node was finally classified as 0. For inconsistencies between 5 minutes and 25 minutes (one is 1 and the other -1) the effect was classified as NaN.

- 1. Gene Expression level

Gene Expression level $x_{Gi,j,k}$of gene *Gi* (G*i* ∈ {1…60}) was measured in 2 or 3 replicates *k* (*k* ∈ {1,2,3}) per stimuli *j* (*j* ∈ {1..26}) at one time point (*t*=360 minutes). The stimuli were distributed on four batches *b* (*b* ∈ {1,2,3,4}; the corresponding batch for stimuli *j* is indicated by *b(j)*) with controls for each gene for each batch. For each batch, we used the sample mean ($X_{Gi,b(j)}$) and the sample standard deviation ($\sigma_{Gi,b(j)}$) of all measurements of *Gi* from this batch to generate the z-score:

$$Zscore_{Gi,j,k}=(x_{Gi,j,k}-X_{Gi,b(j)})/\sigma_{Gi,b(j)}$$

If the z-score was bigger/smaller than a threshold (here: thr=1/-1) we assumed a significant positive/negative effect. In addition to the protein data, expression data of significant effects were excluded from the z-score calculation, i.e. the z-score was re-evaluated (by excluding expression data that showed an consistent effect in *j*) until no significant regulation was found. Furthermore, effects were only considered, if at least 2 of 3 or 1 of 2 replicates were changed. Positive/negative changes were classified as 1/-1 effects. Consistent unchanged nodes or nodes with just one significant change (in case of 3 replicates) were classified as 0. If there was at least one positive and at least one negative effect in replicates for the same stimuli, the effect was inconsistent and thus classified as NaN, i.e. the measurement is not accounted for.

- 1. Cytokine level

Cytokine level of cytokine was measured in one of the 2 batches in 3 replicates per stimuli *j*. Because data from cytokine levels were already provided as a z-score, we just tested the data set on the threshold (here: thr=1/-1) and selected consistent up-/downregulation or unchanged effects, if 2 out of 3 replicates showed the same behavior. As for the other data, inconsistent effects were classified as NaN. Every batch was processed separately.

2. Method for detecting and removing inconsistencies between discretized experimental data and network topology

Our method to improve the agreement between experimental data (the latter given as -1/0/+1 as described in the first step) and the reference network topology is based on the concept of ***sign-consistency***. The key idea is that the change of a measured node in response to a perturbation (e.g., adding a ligand) must be explainable by the direction of change of one of its preceding nodes in the reference network. In cases where inconsistencies between network structure and discretized data are detected, edge additions and removals are used to minimize the mismatches (for details see (Mela*s et a*l., 2013)).

We also developed a software tool (*SigNetTrainer* for MATLAB/CPLEX) which was used herein with some minor modifications to deal with the particular size of the problem at hand.

Assume we are given a signed, directed graph (interaction graph) $G=(V,E,\sigma)$, where *V* is the set of nodes, *E* the set of edges, and *σ* the set of signs corresponding to edges in *E* ($\sigma_{e}\in\left\{-1,1\right\}, e\in E$). We define a sign pattern ***s*** as a node labeling storing for each node *X* a sign $s_{X}\in\left\{-1,0,1\right\}$. The labeling ***s*** is sign-consistent with respect to the interaction graph if the following conditions hold for each node X:

1. If $s_{X}=-1$: there is a predecessor node *Y* and an edge *e*: *Y* -> *X* with $\sigma_{e}\cdot s_{Y}=-1$, or *s_x_* was fixed to *-1* (perturbed node)
2. If $s_{X}=1$: there is a predecessor node *Y* and an edge *e*: *Y* -> *X* with $\sigma_{e}\cdot s_{Y}=1$, or *s_x_* was fixed to *1* (perturbed node)
3. If *s_x_* = 0: either (i) *s_x_* was fixed to 0, or (ii) *X* has no predecessor, or (iii) for all edges *Y* -> *X* we have *s_x_ = 0*, or (iv) there is an edge *e* : *Y* -> *X* with $\sigma_{e}\cdot s_{Y}=-1$ and another edge
   *h* : *Z* -> *X* with $\sigma_{h}\cdot s_{Z}=1$ .

As in the provided experiments only positive perturbations (ligand stimulations) have been performed, the case that a node’s state was fixed to -1 does not occur.

Given a single experimental scenario, we fix the state of the node corresponding to the stimulus to 1 and then search for a sign-consistent node labeling in the given network showing a minimal mismatch with the discretized measurements. In this way, we get for each experimental scenario a fitting error *E*, that is, the sum of the absolute differences

*E* =$\sum_{X:m_{X}\neq NaN} \left|m_{X}-s_{X}\right|$ (1)

between the measurements *m_x_* and the optimal sign pattern *s* (“optimal” meaning that no other sign pattern being consistent with the network topology could lead to a smaller mismatch with the data). Of course, in the ideal case, the error *E* in eq. (1) will be zero.


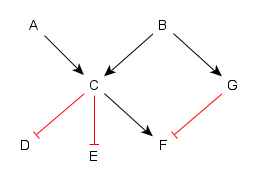
Figure 1: Example Network.

|  | **Stimulus** | | **Measurements** | | | | | **Fitting Error *E*** |
| --- | --- | --- | --- | --- | --- | --- | --- | --- |
|  | **A** | **B** | **C** | **D** | **E** | **F** | **G** |  |
| **Exp. 1** | 1 | 0 | 1 | 0 | -1 | 1 | NaN | 1 |
| **Exp. 2** | 0 | 1 | 0 | 1 | 0 | -1 | NaN | 4 |

Table 1: Example Scenarios for example network (Fig. 1). NaN indicates that no measurement was available.

As an example, consider the small network given in Fig. 1 and assume we measured the nodes C, D, E, and F (G is not measured an thus a latent node) in response to stimulation with A (Exp. 1) or B (Exp. 2). The measurements are given in Table 1. For experiment 1, all measured states except the state for D are in accordance with the following sign-consistent node labeling (obtained as prediction from the network topology): C=1, D=-1, E=-1, F=1, G=0. Thus, we get a fitting error of 1 for this experiment (D should be -1 but was measured to be 0). For experiment 2, the optimal sign-consistent node-labeling is C=1, D=-1, E=-1, F=-1, G=1. Here, the measured states of three nodes disagree: C and E were measured unchanged, but, according to the network topology, C should increase and E should decrease. Thus, both species add the value 1 to the fitting error. For D, the mismatch is even larger: an increase was measured, although a decrease would have been expected. In this case, the value 2 is added to the fitting error. Hence, a fitting error of 4 results for the second experiment. The cumulative fitting error over all scenarios is thus 5.

Based on these fitting errors, we now try to optimize the network structure in order to minimize the fitting error over all scenarios. In a first step, we only allow the removal of edges. In our example network (Fig. 1 and Table 1), removing the edges C --| D and B --| C improves the fitting error over all scenarios from 5 to 1: according to the new network structure, the sign-consistent labeling in Exp. 1 is equal to the measurements (i.e. the fitting error is 0); for Exp. 2, the optimal sign-consistent labeling is C=0, D=0, E=0, F=-1, G=1 (i.e. the fitting error has been reduced to 1).

In general, more than one optimal solution for removing edges exists. In particular, if the measurements are rather sparse compared to the network size, several sub-networks might all explain the data equally well. Ideally, one can enumerate all these optimal solutions. However, the large network size of the given network in the sub-challenge makes this impossible. Moreover, submission of multiple solutions was not allowed in this sub-challenge.

Therefore, we had to restrict ourselves to compute only one optimal solution. We chose a solution where a minimal number of edges is removed. However, even such an approach can lead to non-unique solutions. For example, in a linear chain S🡪A🡪B🡪C🡪D🡪M with stimulus S and measured node M (and latent nodes A,B,C,D) we might have the case that stimulating S did not lead to increase of M (M=0). Our method would then remove exactly one of the 5 edges (to interrupt the signal flow), however, it represents a non-unique solution. Here one would need more measurements to resolve such ambiguities. This should be kept in mind when interpreting the results. Sometimes, a well-established (“certain”) edge might be removed in the reference network. At least in some cases, this might be due to multiple solutions and other solutions might exist where “less” evident edges are removed. However, to get an unbiased result, we did not order the edges with respect to their evidences (but this would, in principle, be possible).

From the example in Fig. 1 / Table 1, we see that it might be necessary to consider not only the removal, but also the addition of edges. In Exp. 2, the measurements indicate that there is an additional positive effect from B to D not running over C. Thus, adding the edge B 🡪 D (or, as equivalent solutions, F --| D or G 🡪 D) would remove the fitting error completely.

Edge additions are done in a second step (after network pruning described above). However, the search space for edge additions (i.e. the number of possible edges) increases dramatically with the network size (in the given reference network ~ 100,000 different (directed and signed) edge candidates exist), so that finding a *globally optimal* solution for edge additions is, in general, not possible in large networks. In a heuristic approach, one can use a greedy algorithm: in the first iteration, search for the edge that decreases the error value the most (by exhaustively testing all single edges) and add this edge to the network. In subsequent iterations, this is repeated again and again leading after *n* iteration to the addition of *n* new edges. The algorithm stops if no further significant improvement can be achieved by addition of a single edge. However, even this simplified approach is computationally demanding (see also below). The main reason is that finding an optimal sign-consistent pattern for a given network topology and experimental scenario (with measurements) is not trivial. In *SigNetTrainer*, we use an Integer Linear Programming implementation (ILP) (i) to encode sign-consistency, (ii) to search for (globally) optimal edge deletions minimizing the network-measurement-mismatch over all scenarios, and (iii) to search via a greedy algorithm for most effective edge additions. The ILP problem is solved via CPLEX.

3. Pseudo-code of the whole procedure:

- - 1. *Data processing / Discretization*
       - - calculate z-score for protein phosphorylation data, for every batch separately
- identify effects in z-score by threshold
- need 2 of 3 effects in replicates for a relevant -1/+1 change
- remove effects with inconsistent signs in replicates and between 5 and 25 minutes
- calculate z-score for gene expression data, for every batch separately
- identify effects in z-score by threshold and new calculation of mean and standard deviation iteratively
- 2 of 3 or 1 of 2 replicates significant for a relevant -1/+1 effect
- remove effects with inconsistent signs in replicates
- identify effects in z-score of cytokines by threshold
  - - - - need 2 of 3 effects in replicates for a relevant -1/+1 change
        - remove effects with inconsistent signs in replicates
    1. *Use classified datasets to detect and remove inconsistencies between experimental data and network topology by edge removals/additions*
- Search for (globally) optimal set of edge deletions that minimizes the error between sign-consistent predictions and discretized measurements over all scenarios.
- Use afterwards greedy algorithm to search for (locally) optimal edge additions (one edge in each iteration) minimizing the error between sign-consistent predictions and discretized measurements over all scenarios.

4. Some remarks on the results

- We used the SIGNED version of the reference network which was also provided on the SBV IMPROVER website. We assumed a positive edge sign if no sign was provided.
- Given the reference network and the discretized data, the error value *E* in eq. (1) (cumulated over all scenarios) was 1089 for the human data and 1073 for the rat data.
- Searching for globally optimal edge deletions: with the very large reference network and the measurements from 26 experiments, this becomes a huge optimization problem with more than 100,000 constraints on ~80,000 integer variables. We stopped the search with a timeout of 6 hours (this is possible in CPLEX; the best solution found until the time out is then delivered). It might thus be possible that the solution found for human / for rat are not globally optimal yet. However, the large reduction in the cumulated error value indicates that we are probably at least close to an optimal value: In case of the human data, the suggested edge deletions (~ 210 edges) reduced the total fitting error from 1089 to 346; in case of the rat data the suggested deletion of ~ 190 edges leads to a reduction in the cumulated fitting error from 1073 down to 337.

(Note that there were two parallel edges in the reference network; we included only one of both in the final network as we can anyway not distinguish them.)

- Most of the suggested edge deletions refer to edges with genes as targets, hence, these links seem to be ill-defined. Furthermore, several edge deletions disconnect certain inputs from the rest of the network, such as Betahistine🡪PLC; Norethindrone🡪PGR_family; AREG🡪EGFR (these three edges were even deleted in both human and rat network). Those deletions indicate that the majority of targets in the networks were not responsive to the respective stimulus.
- As described in section 2, after edge deletions we used a greedy algorithm to search for suitable edge additions that would decrease the cumulated fitting error. For biological plausibility, edge additions were not allowed (i) from cytokines to any other node, (ii) from genes to other nodes except to cytokines as well as (iii) if the edge would end in an input node. We used a threshold *tred_err*=3 stating that an edge addition must decrease the cumulated fitting error at least by three. Somewhat surprisingly, in the end, only two (human) and four (rat) such edge additions with significant error reduction could be found reducing the cumulated fitting error to a final value of 336 (human) and 321 (rat), respectively. Interestingly, for both human and rat we found that a link connecting PROMETHAZINE with some region of the network is missing. Note that, as in the case of edge removals, several equivalent solutions for an edge addition might exist. For example, the added link PROMETHAZINE🡪GRB2 in the rat network could be exchanged by PROMETHAZINE🡪SOS or PROMETHAZINE🡪RAS (and even others) pointing again to non-determinacy.

**Teams 116 & 93** (Lujia Chen, Xinghua Lu)

We addressed this task as learning species-specific Bayesian network that provides the best representations of the observed data based on a reference network as prior knowledge. A Bayesian network is a directed acyclic graph (DAG) to represent the joint distribution of a set of variables, and with certain constraints it can represent the causal relationship among these variables. *De novo* learning structure based on observed data is NP-hard, which involves searching for a super exponential space of possible structures, but prior knowledge helps to constrain the searching space. Given the regulatory relationships between these nodes, a Bayesian network can efficiently represent the regulatory effects of the upstream nodes on the downstream ones based on conditional probability. Therefore, the task can also be considered as searching for the variants of the reference network that can best explain the observed data.

In our setting, the phosphorylation states of proteins and expression states of genes represent the nodes in a Bayesian network, and a directed edge between a pair of nodes represents the regulatory (causal) relationship between variables.  Each observed node takes a binary state (0/1); unobserved variables are represented as latent variables in Bayesian network. We augmented the reference network by adding edges base on our knowledge, and used the augmented graph as initial candidate network structure. We further defined the conditional probability between a node and its parent nodes as logistic function. We then employed a Markov chain Monte Carlo (MCMC) algorithm to infer the states of latent variables, and estimate the parameters associated with the edges using elastic network regression.  This method enabled us to optimize the computation cost and search for a sparse Bayesian network that well modeled the data.

**Team 83** (Anastasia Chasapi, Ioannis Xenarios, Leonore Wigger, Julien Dorier, Mark Ibberson, Nicolas Guex)

For the Species Specific Network Inference Sub-Challenge of SBV Improver, we decided to conduct a treatment-based analysis. The goal was to identify the genes that are differentially expressed in each treatment, and create treatment specific networks, based on the provided reference network. The ensemble of all treatment specific networks for a species constitutes the species-specific network. All scripts of this work were developed in R, and several packages were used, such as limma and igraph.

1. Data Mapping

The first step of the analysis was to identify the genes that are present in the reference network but do not appear with the same name in the gene expression data. The NCBI gene database was queried for those genes and the gene expression datasets were scanned against the alternative names retrieved. In case there was more than one matching measurement in the data, the most variant gene would be selected. All cytokine and phosphoprotein names were mapped to their gene names using UniProtKB. Thereafter, all data files were filtered to contain only elements found in the reference network.

2 Data Normalization

The workflow in its entirety was performed separately for the human and rat measurements. During the first step of normalization, each batch was treated separately. The average control (DME) value of all repetitions from the specific batch was calculated for each gene. The average values vector was then subtracted from the treatment expression values belonging to the same batch. No averaging was performed for treatment repetitions.

For the second normalization step all data were merged. For each repetition of each treatment the z score was calculated. The same process was followed for the phosphoprotein and cytokine datasets.

3 Treatment Specific Differential Expression

To define treatment specific, differentially expressed genes, we performed a moderated t-test for each treatment against the remaining treatments and controls, and selected genes with p value < 0.05 as differentially expressed. This process was repeated in all datasets (gene expression, phosphoproteins, cytokines). ANOVA analysis of the gene expression dataset revealed a number of genes that were not found with the t-tests. We assumed that this is a result of treatments that have very similar expression patterns but their effect is underestimated when one of the treatments is used in the “control set” for the t-test against the other. We used hierarchical clustering on the treatments, and divided them to clusters to test this hypothesis. We performed again the t-test for each treatment against the rest, excluding members of the same cluster. Indeed, this method yielded additional differentially expressed genes that were added to the results.

4 Treatment specific network inference

At this stage we had a list of highlighted (i.e. differentially expressed) genes for each treatment, derived from t-tests performed in gene expression, phosphoproteins, cytokines and GEx clustered datasets. For the network inference of each stimulus, we evaluated the reachability of every highlighted gene pair in the reference network. Having as a principle that every highlighted gene should be reachable from the stimulus node, we added missing edges according to the following rules:

1. Every node should be reachable from the stimulus node
2. If not, test if the node is connected to other upstream, highlighted nodes
3. If it is not connect node to all highlighted nodes of the level above
4. If none, reveal the paths of all upstream nodes to all downstream nodes, and connect the node to all nodes of the level above.

**
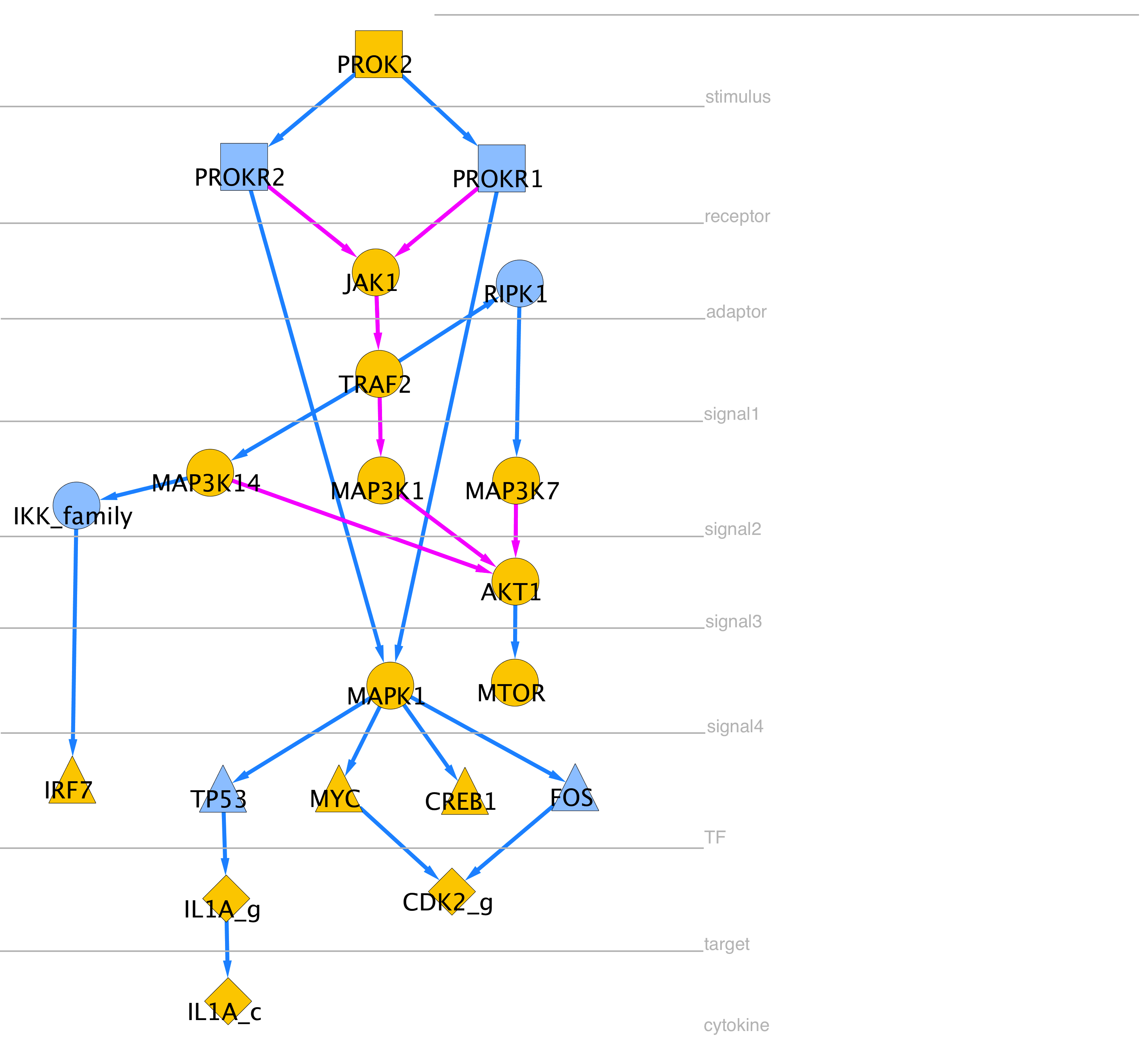
**

Adding missing links for treatment specific network inference, the case of human PROKINECITIN2. Yellow nodes are differentially expressed for PROK2 and blue nodes are not, but are used for path reconstruction. Blue links represent already existing paths and pink links are the ones added. In the case of JAK1, there were no highlighted nodes on the level above. Therefore, all paths that connect PROK2 to downstream nodes were revealed and JAK1 was connected to the nodes of the level above, i.e. PROKR1 and PROKR2. For the connection of TRAF2 no path revealing was necessary since JAK1, which is on the level above, was already highlighted. The same principles are applied for the rest of the added edges.

5 Species specific network inference

The added edges of all treatments were appended to the reference network. For every highlighted gene pair of each stimulus, all shortest paths were calculated. Every time an edge was found in the path, the edge received +1 point (which corresponds to number of visits). The final, species-specific network, was derived by keeping only the visited edges of the extended reference network

**Team 104** (Hugh Mitchell, Joel G. Pounds, Susan Tilton, Jason McDermott)

1. Initial processing

Non-conventional node names in the reference network were manually matched to the identifiers used in the provided data. Multiple matches for a single nonconventional ID resulted in an expansion. This process caused the reference network to grow from 501 unique edges to 1236 for the human network and 1159 for the rat. This conversion and expansion allowed for inferences made from the provided data to be mapped back to the reference network. In addition to these changes, replicate treatments were collapsed to average values in all data sets, and genes with no detectable signal were removed to facilitate the prediction process.

2. Identification of candidate regulatory interactions based on ligand treatments

Candidate interactions could be identified by tracing signaling paths through the network to key effectors, and then building edges between these effectors and the genes/proteins observed to be perturbed in response to ligand. For gene expression data, key effectors are transcription factors (TFs); for phosphorylation they are kinases and phosphatases. Genes, cytokines and phosphoproteins changed to a statistically significant degree in response to each ligand treatment were identified using standard ANOVA analysis. A network traversal algorithm was implemented in which the expanded reference networks were surveyed in a series of traversals, each beginning at a different ligand stimulus point. For each traversal, each downstream node was visited only once, and each encountered node that was identified as a key effector was logged for each ligand. Candidate edges were then built between 1) the key effectors downstream of each ligand, and 2) the genes/proteins observed to be affected by that ligand. Significantly changed cytokines were treated as alternative gene expression readouts, thus potentially redundant candidate edges were built when data for both a gene and its cytokine product were present.

3. Edge inference

For network inference using the Inferelator software, we used the ‘BBSR’ setting, with the reference network and candidate edges described above as priors, with a prior weight of 2.0. Cytokine measurements were incorporated alongside the gene expression data as if they were additional genes. Input to the inference process was limited to regulators and targets present in the expanded reference networks. Phosphorylation-based interactions were inferred in a similar manner, using known kinases and phosphatases as candidate regulators in place of TFs, and phosphorylation data in place of transcriptional data. Each of the two supplied time points were inferred as independent conditions; an edge inferred from either or both time points was counted as a valid interaction.

4. Network cleanup

To associate inference predictions with a statistical confidence measure, we permuted gene, cytokine and phosphoprotein expression values and performed inference over 500 runs. This approach identified regulatory elements whose expression patterns cause them to be promiscuously incorporated in inference predictions, giving them a high background of incorporation in random data sets. Predicted edges were eliminated which were observed with significant frequency (>5%) in permuted data. In addition, regulators whose overall incorporation rate did not change in a significant portion (>20%) of the permutations when compared to real data, were removed from the inference. In addition, edges were removed for which the expression patterns of the regulator and target were inconsistent with the known enzymatic activity of the regulator (correlation < -0.2 for kinases, > 0.2 for phosphatases). The two sets of phosphorylation-based edges (from the two time points) and the transcription-based edges were combined into one set of unique inferred edges for each species. Edge IDs were then collapsed to the nomenclature used in the original reference network, using a reversal of the initial expansion step. Inferred edges present in the original reference network were removed from one species’ network only if the inference procedure supported their presence in one species, but not the other. If the existing edge were not predicted by either inference procedure, the edge was retained.

**Team 50** (Sahand Hormoz, Gyan Bhanot, Adel Dayarian, Michael Biehl)

To correct for hybridization saturation in gene expression levels, we generated a universal noise curve from variation in replicates under the assumption that expression level measurement noise is only a function of the mean expression level and is independent of the type of gene considered. Outlier measurements were removed if they were more than three standard deviations from the mean using this universal noise curve. Since the phosphorylation data had no saturation we used the replicates directly to estimate noise. A strict t-test p-value cutoff of p<0.01 was used in case/control comparisons to identify informative genes or proteins for each stimulus. Details of the processing of gene expression and phosphorylation data are given in (Hormo*z et a*l., in press).

To generate the network for each stimulus, we used the connectivity of the reference network and analyzed the data for each stimulus separately. For each stimulus, we form a sub-network by removing all other stimuli and including only the nodes that are connected to the considered stimulus. A node that is activated by the stimulus is designated as ON, otherwise it is designated as OFF. Each stimulus provided us with evidence for or against the existence of each edge in the network. This evidence was in the form of a sequence of positive and negative votes. For each edge we cast one positive vote if it connects an ON node to another ON node. Similarly, we cast one negative vote if the edge connects an ON node to an OFF node. We combined the votes from all the stimuli to infer the overall network. Finally, we trimmed the network by removing edges with only negative votes. We also added edges such that the ON nodes which are not originally connected to the sub-network of a stimulus become connected.

**Team 131** (Jie Cheng)

1. Data preprocessing

Within each batch, we averaged the repeats and subtracted mean control value. If the value is smaller than a threshold, we set the value to zero. We used threshold 0.7 for gene expression, cytokine and phosphoprotein data. For gene expression data, we removed genes that have more than 21 zeros under 26 stimulus conditions. For phosphoprotein data, we combined 5 min and 25 min data by taking the value that had larger absolute value. We combined data from all three platforms and selected variables that were included in the reference network.

2. Network learning

We implemented the network learning algorithm described in (Chen*g et a*l., 2002). We run bootstrap 100 times and counted the frequency of each edge. The final network was generated by modifying the reference network – an edge was removed if both variables were in our data and the edge did not appear in our bootstrap runs; if an edge appeared in more than 40% of the bootstrap runs, we added it to the network.

**Team 70 (**Boris Breuer, Huub ten Eikelder, Dragan Bošnački, Peter Hilbers)

Our method determines the presence of an edge between two network elements by using a combination of two main criteria: correlation and significance, both expressed as p-values. The first p-value,$p_{z}\left(i,j\right)$, is generated from a z-test,

$$z_{ij}=\left|\left(m_{ij}-\mu_{j}\right)/\left(\sigma_{j}/\sqrt{n}\right)\right|$$

where $m_{ij}$ is the measurement of the considered factor *j* (gene, phosphoprotein or cytokine) under influence of the factor *i*, and $\mu_{j}$ and $\sigma_{j}$ are respectively the mean and standard deviation of the measurements of factor *j*. The values of $\mu_{j}$ and $\sigma_{j}$ have been computed from all available measurements of factor *j* except the one under influence of factor *i* (Pinn*a et a*l., 2010).

The second p-value, $p_{d}\left(i,j\right)$, is generated from a distance covariance (Székely and Rizzo, 2009) permutation test between all measurements of factors *i* and *j*. Moreover, to each edge that occurs in the reference network a third scalar p-value, $p_{r}$, is assigned. To obtain this constant value $p_{r}$ a non-decreasing list, ***p*,** of all $p_{z}\left(i,j\right)$ and $p_{d}\left(i,j\right)$ values is constructed. In ***p*** we choose the element at position $\left\lfloor length\left(p\right)\cdot x\right\rfloor$, where *x* is the fraction of all possible edges that actually occur in the reference network, i.e. $p_{r}$is the x^th^ quantile of ***p***.

The two (or three) p-values are multiplied to eventually obtain a final p-value by employing the product distribution of n uniformly distributed random variables, similar to Fisher's combined probability test. The edge is considered part of the network if its p-value is below a final threshold value of 0.01. This procedure is repeated for both rat and human networks.

# references

Cheng,J. *et al.* (2002) Learning Bayesian networks from data: An information-theory based approach. *Artif. Intell.*, **137**, 43–90.

Hormoz,S. *et al.* (in press) Inter-species Inference of Gene Set Enrichment in Lung Epithelial Cells from Large Proteomic and Transcriptomic Data Sets. *Bioinformatics*, **this volume**.

Lalaker,A. *et al.* (2009) Chitin stimulates expression of acidic mammalian chitinase and eotaxin-3 by human sinonasal epithelial cells in vitro. *Am. J. Rhinol. Allergy*, **23**, 8–14.

Lamb,J. *et al.* (2006) The Connectivity Map: Using Gene-Expression Signatures to Connect Small Molecules, Genes, and Disease. *Science*, **313**, 1929–1935.

Melas,I.N. *et al.* (2013) Detecting and removing inconsistencies between experimental data and signaling network topologies using integer linear programming on interaction graphs. *PLoS Comput. Biol.*, **9**, e1003204.

Mertens,V. *et al.* (2010) Gastric juice from patients ‘on’ acid suppressive therapy can still provoke a significant inflammatory reaction by human bronchial epithelial cells. *J. Clin. Gastroenterol.*, **44**, e230–235.

Pinna,A. *et al.* (2010) From Knockouts to Networks: Establishing Direct Cause-Effect Relationships through Graph Analysis. *PLoS ONE*, **5**, e12912.

Poussin,C. *et al.* (2014) The Species Translation Challenge – A Systems Biology Perspective on Human and Rat Bronchial Epithelial Cells. *Sci. Data*, **1**.

Székely,G.J. and Rizzo,M.L. (2009) Brownian distance covariance. *Ann. Appl. Stat.*, **3**, 1236–1265.

Zhijin Wu,R.A.I. (2004) A Model-Based Background Adjustment for Oligonucleotide Expression Arrays. *J. Am. Stat. Assoc.*, **99**, 909–917.
